# Reversible Magneto-Ionic Control of Exchange Bias in Coupled Spin-Valve-Like Heterostructures

**DOI:** 10.1021/acsami.5c10187

**Published:** 2025-08-24

**Authors:** Markus Gößler, Jonas Zehner, Rico Huhnstock, Falk Röder, Rico Ehrler, Olav Hellwig, Arno Ehresmann, Karin Leistner

**Affiliations:** 1 Institute of Chemistry, Chemnitz University of Technology, Chemnitz 09107, Germany; 2 Leibniz Institute for Solid State and Materials Research, Dresden 01069, Germany; 3 Institute of Physics, University of Kassel, Kassel 34132, Germany; 4 Center for Interdisciplinary Nanostructure Science and Technology (CINSaT), 214794 University of Kassel, Kassel 34132, Germany; 5 Leibniz Institute of Polymer Research, Dresden 01069, Germany; 6 Institute of Physics, Chemnitz University of Technology, Chemnitz 09107, Germany; 7 Center for Materials Architectures and Integration of Nanomembranes (MAIN), 38869 Chemnitz University of Technology, Chemnitz 09107, Germany; 8 Institute of Ion Beam Physics and Materials Research, Helmholtz-Zentrum Dresden-Rossendorf, Dresden 01328, Germany

**Keywords:** magneto-ionic, exchange bias, spin valve, interlayer coupling, GMR

## Abstract

Voltage control of exchange bias (EB) is an important technological goal for low-power spintronic sensor and memory devices. The magneto-ionic (MI) approach for voltage-controlled EB is a promising strategy to achieve this goal, utilizing electrochemical reactions at low operational voltages. In typical MI devices, however, the sensitive EB layers are directly targeted by the electrochemical reactions, which often impairs reversibility. Here, we introduce an alternative device structure by isolating the EB layers from the active MI layer. Making use of the interlayer (IL) coupling through a spacer layer in an IrMn/Fe/Au/Fe spin-valve-like heterostructure, we show that EB can be reversibly controlled by an electrochemical modification of the top layer. Using the same device structure, we also realize an MI switching between single-step and double-step hysteresis loops. We interpret the observed MI effects via an increasing top Fe layer thickness, caused by the electrochemical reduction of FeO_*x*_ to ferromagnetic Fe. Modeling of hysteresis loops as a function of top layer thickness in an extended Stoner–Wohlfarth approach corroborates this interpretation. Our results highlight an advanced strategy for improving reversibility in MI devices and open a novel pathway toward voltage-controlled spin valves.

## Graphical abstract

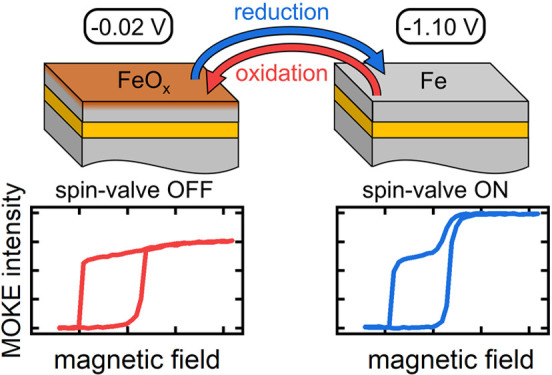

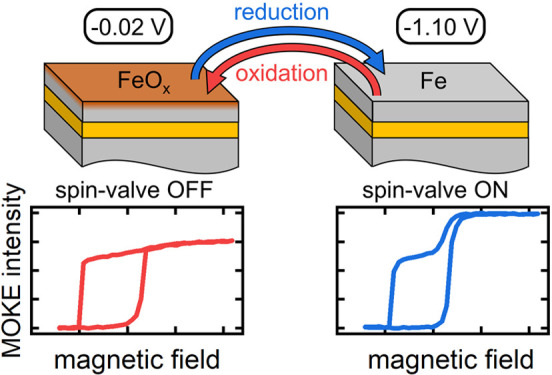

## Introduction

Exchange bias (EB) is a phenomenon that arises at the interface of a ferromagnet (FM) and an antiferromagnet (AFM), resulting in a shift of the FM’s hysteresis loop along the field axis.^1,2^ It can be described as a unidirectional anisotropy contribution acting on the FM, which has its origin in the exchange interaction of spins across the FM/AFM interface. The EB anisotropy is usually set by field cooling^1^ but may also be induced by field growth,^3^ thermally induced ordering,^4^ or ion bombardment in a magnetic field.^5^ In a classical EB system, AFM spins thereby align with the ordered spins at the FM interface and the EB anisotropy direction is set in the direction of the applied field during field cooling, field growth, or ion bombardment causing a loop shift in the opposite field direction (negative EB). Controlling this EB anisotropy after field cooling at a fixed temperature is an important goal of contemporary research, which could enable further advances in the technological application of the EB effect in magnetoresistive sensing,^6,7^ antiferromagnetic spintronics,^8,9^ and magnetic particle transport (magnetophoresis).^10,11^

A promising path achieving this goal is voltage control of magnetism. Within this field, various device concepts have been explored specifically for the voltage control of EB, with the most prominent examples being magnetoelectric/multiferroic,^12,13^ strain-mediated,^14,15^ resistively switchable,^16,17^ and magneto-ionic (MI) devices. MI approaches rely on chemical changes within the magnetic material either by (electro-)­chemical reactions or ion migration. They are advantageous over other approaches in terms of the nonvolatility of the magnetic changes, their low operational voltage, and their overall energy efficiency.^18^ These factors, as well as the application potential in prospective fields such as neuromorphic computing^19^ and spintronics,^20^ have caused a boom in MI research over the past decade.^18–21^

As a result, several MI mechanisms for the control of EB have also been reported in recent years. Ion migration without a voltage, i.e., solely driven by chemistry, has been shown to impact EB after device preparation.^22–26^ For voltage-controlled EB, MI devices have been gated in either solid^27–31^ or liquid electrolytes.^32–37^ In such MI devices, typically either FM^29,33,37^ or AFM layers^27,30,32^ are in direct contact with an ionic reservoir or the electrolyte solution, enabling the electrochemical modification of either FM or AFM layer by ion migration or electrochemical reactions. Thereby, MI control of EB has been effectively demonstrated in a variety of AFM/FM combinations. However, such MI switching schemes for EB also have a recurrent limitation, in the form of partly irreversible effects during MI treatment.^28,31–33^

Such irreversible effects have previously been ascribed to the degradation of the EB interface by electrochemical reactions.^32^ Although direct electrolyte contact of the EB interface is generally prevented in MI device geometries by either a closed FM or AFM layer in contact with the electrolyte, electrochemical reactions may still occur at the EB interface. This could happen, e.g., via solid-state diffusion of involved ions or via electrolyte infiltration of the contact layer through capillaries at the grain boundaries.^38^ Besides interface degradation, the emergence of structural or chemical defects at the EB interface during (repeated) electrochemical reactions could also affect reversibility for the MI control of EB. It is known that EB is highly sensitive not only to magnetic training effects^39^ but also to the structural quality of the interface and chemical changes. Lattice defects (vacancies^40^ or dislocations^41^), grain size,^42,43^ and crystalline quality^44^ in AFM or FM layers are known to be important factors affecting the EB. Layer roughness^45^ and layer intermixing^39,40^ at their interfaces can also have a pronounced impact on the EB. All these factors could be influenced once electrochemical reactions occur at the EB interface, leading to irreversible contributions to MI effects. Here, we aim to mitigate such irreversible MI contributions and thus improve reversibility in the MI control of EB by multilayer engineering. This is accomplished by isolating both EB layers from the active MI layer in contact with the electrolyte using a noble metal interlayer (IL) in an AFM/FM/IL/FM multilayer design. Such a sequence with a nonmagnetic IL separating two FM layers represents a spin-valve heterostructure.

Our spin-valve-like heterostructures are based on the specific EB system IrMn/Fe. While IrMn is an industry-relevant AFM layer, natively oxidized Fe thin films exhibit a low-voltage MI effect in alkaline electrolytes.^46–48^ This MI effect in Fe thin films is based on the (partial) electrochemical reduction of the native FeO_*x*_ surface layer to metallic Fe, which changes the thickness of the Fe layer. Previous results on Fe thin films have shown that the thickness of such a native FeO_*x*_ surface layer is between 2 and 3 nm.^33,46,47^ Considering the volume expansion during oxidation, this corresponds to the oxidation of only 1.5 nm of the Fe layer,^48^ which can potentially be recovered by electrochemical reduction. The MI control of coercivity,^46^ magnetoresistance,^47^ and magnetization^48^ has been demonstrated using this mechanism, while it has also been applied to control EB in the IrMn/Fe system before.^33^ However, MI reversibility was also limited in this EB system.

In the present work, EB IrMn/Fe layers are locally separated from a top Fe/FeO_*x*_ layer by a nonmagnetic Au IL. The Au IL blocks electrochemical reactions from the EB-pinned Fe layer and thus protects the sensitive EB interface at the same time. We show that the voltage-induced electrochemical reduction of the native FeO_*x*_ layer on the surface of the top Fe layer into metallic Fe leads to an increase in overall top Fe layer thickness. This thickness increase not only influences the magnetic properties of the top Fe layer but also affects the EB-pinned Fe layer and thus allows controlling EB indirectly by IL coupling through the Au IL. This approach leads to a significantly improved MI reversibility compared to IrMn/Fe/FeO_*x*_ structures. Besides the electrochemical control of EB in IL-coupled spin-valve-like structures, we also provide a proof-of-concept demonstration for the voltage control of spin-valve functionality by the MI effect using the same IrMn/Fe/Au/Fe/FeO_*x*_ heterostructure. We show that a reversible toggling between single-step hysteresis loops and double-step hysteresis loops can be achieved in this system. This not only presents a new method to achieve tunable sensitivity in magnetic field sensors but also could initiate voltage-controlled MRAM devices in the future.

## Results and Discussion

### Layer Structure and Magnetism of Coupled Spin-Valve-Like Heterostructures

The schematic architecture of our in-plane magnetized Au/IrMn/Fe/Au/Fe heterostructures is illustrated in Figure 1a. A 5 nm Au underlayer serves as a seed for the textured growth of a 30 nm IrMn (Ir_17_Mn_83_) antiferromagnetic layer. At the interface to the neighboring 10 nm Fe ferromagnetic layer, EB is induced by the application of an in-plane field during sputtering (field growth). Layer thicknesses of the IrMn and lower Fe layer are selected to obtain a strong EB effect.^49^ On top of the Fe layer, a nonmagnetic 4 nm Au spacer layer is grown in order to mediate IL coupling to a second ferromagnetic Fe layer on top with varying thickness *t*_top,nom_. Overall, our layer sequence represents an exchange-biased spin-valve-like heterostructure. Upon air exposure after sputtering, a thin native FeO_*x*_ layer forms on the top Fe layer surface as sketched in Figure 1b, while the Au spacer protects the base layers from oxidation. The voltage-induced electrochemical reduction of this FeO_*x*_ surface layer into metallic Fe provides the basis for the MI effect in our heterostructures.

**Figure 1:**
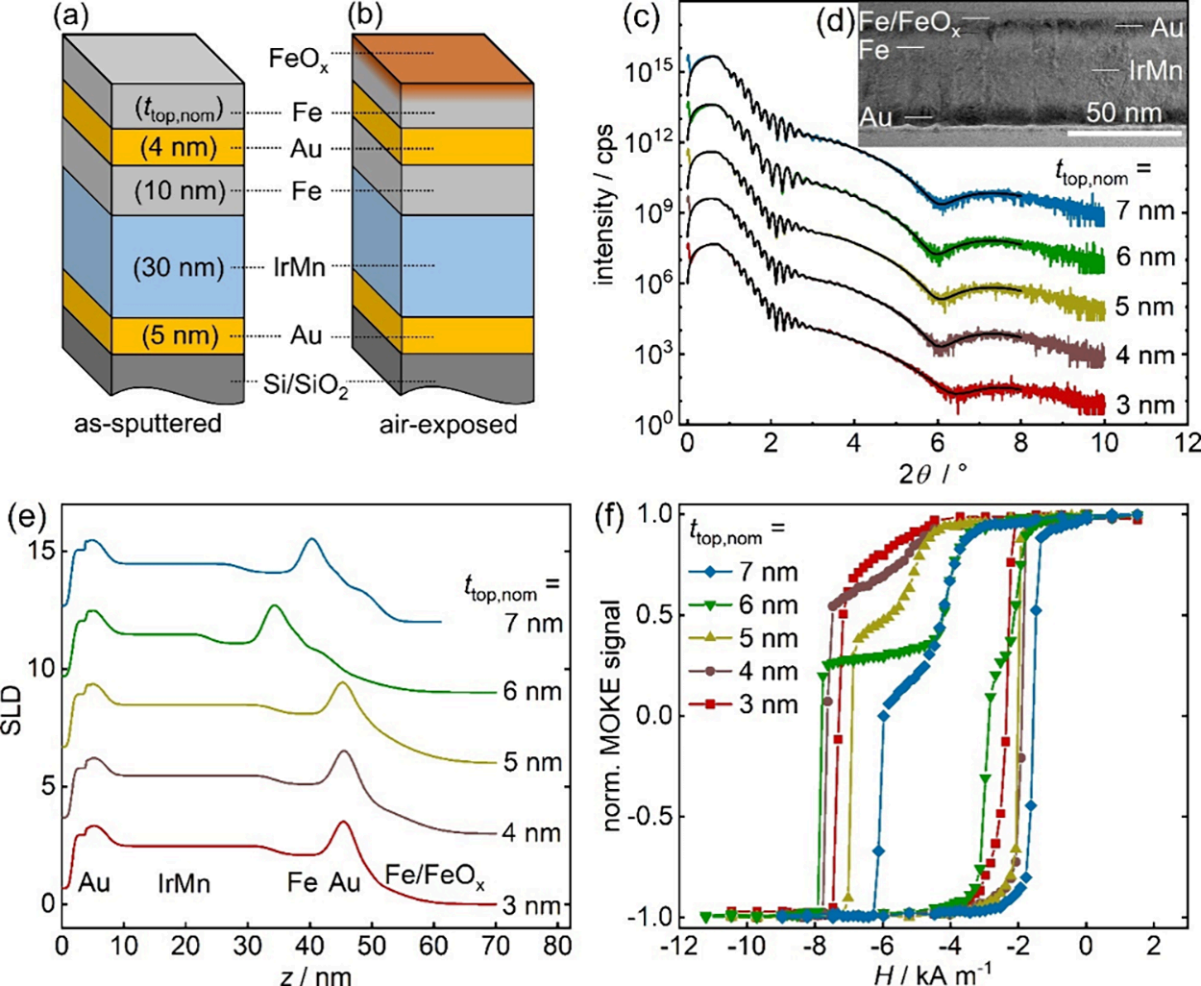
Schematic layer architecture of the coupled Au/IrMn/Fe/Au/Fe heterostructures (a) in the as-prepared state and (b) in the air-exposed state. A fraction of the top Fe layer is transformed into FeO_*x*_ upon air exposure. (c, e) X-ray reflectivity (XRR) data and corresponding scattering length density (SLD) profiles derived from XRR measurements for heterostructures with different nominal FM top layer thicknesses *t*_top,nom_. Fits to the XRR data are shown as black lines. Curves are shifted on the *y*-axis for comparability. Labels close to the *x*-axis in (e) indicate the approximate position of each layer in the SLD. (d) A representative cross-sectional TEM image is depicted for a sample with *t*_top,nom_ = 5 nm. MOKE hysteresis loops of air-exposed samples for different *t*_top,nom_ are shown in (f).

To verify the nominal layer structure, we characterized our systems using X-ray reflectivity (XRR). Scans recorded for all samples, where we changed the nominal thickness of the top Fe layer (*t*_top,nom_ = 3 to 7 nm), are depicted in Figure 1c together with fits to the curves in black. All curves exhibit Kiessig oscillations typical for thin film systems with thicknesses in the range of 1–100 nm, which contain detailed thickness and interface roughness information via their oscillation period and damping. The corresponding scattering length density (SLD) in Figure 1e, which represents the electron density profile of the heterostructure, is derived from fits to the data. Thickness values extracted from the SLD for all layers are given in Table S1 in the Supporting Information.

The two peaks in the SLD correspond to the high electron density of the Au base layer and interlayer. The plateau between the two peaks is attributed to the IrMn AFM layer and the pinned FM layer, the latter causing a small dip in the SLD. Top Fe and FeO_*x*_ layers appear as broad shoulders at large distances *z*, rendering a direct comparison of layer thickness for the topmost layers unfeasible. For the EB underlayers, however, XRR measurements confirm the nominal layer structure with deviations below 10%, with two exceptions: For the samples with *t*_top,nom_ = 6 nm and *t*_top,nom_ = 7 nm, the IrMn AFM layer is significantly thinner (17 and 22 nm) than the intended nominal thickness (30 nm). This results in a narrower SLD profile (green and blue curves in Figure 1e) compared to the other curves. While we cannot resolve the exact reason for this reduced AFM thickness during sputtering, it also influenced the magnetic properties for the sample with the thinnest AFM layer thickness, which are discussed later in this section. Nonetheless, we keep this sample in our series, as it allows demonstrating the robustness of our MI effect with respect to structural deviations.

We additionally characterized our samples using transmission electron microscopy (TEM) and depict a representative cross-sectional TEM image for a sample with *t*_top,nom_ = 5 nm in Figure 1d. Individual layers are labeled in the image. The nominal layer structure is also confirmed for this sample using this real-space method. In the Supporting Information, we additionally show energy-dispersive X-ray spectroscopy (EDS) maps in Figure S1, which confirm the composition of the individual layers. Thicknesses extracted from EDS profiles are given in Table S2 in the Supporting Information, which are in excellent agreement with thicknesses determined using SLD profiles for the measured samples. TEM images reveal that our samples grow in a columnar grain structure with average lateral grain sizes of ∼15 nm, which will be discussed as the main source of magnetic interlayer coupling later.

The effect of top layer thickness on the magnetic properties in our spin-valve-like heterostructures is exemplified in Figure 1f. Hysteresis loops recorded via magneto-optical Kerr effect (MOKE) magnetometry are shown for samples with five different nominal top layer thicknesses *t*_top,nom_ from 3 to 7 nm, while the base of the heterostructure remains unchanged. In general, the measured hysteresis loops are characteristic for spin-valves,^50^ with a shifted EB loop for the pinned FM layer (lower loop) and a second loop for the top FM layer (upper loop) creating a double-step loop in total. For decoupled FM layers, the top layer loop would be centered around zero field, while we observe a significant shift of the top layer loops in the EB direction that indicates a strong ferromagnetic coupling between the two FM layers. For the thinnest top layer (*t*_top,nom_ = 3 nm, red squares), a single-step hysteresis loop with no separate top layer loop is observed, as the strong coupling even allows collective reversal in this case. With increasing *t*_top,nom_, the loop for the top layer becomes more pronounced as a result of its larger magnetic moment. In the field sweep to positive fields, both pinned and top FM layers switch at a common reversal field for *t*_top,nom_ ≤5 nm. Only for *t*_top,nom_ = 6 nm (green triangles) two distinct switching fields are also visible in the field sweep to positive fields, indicating an independent switching of both FM layers in that field direction. For the thickest FM top layer (*t*_top,nom_ = 7 nm, blue diamonds), this second step vanishes again. We attribute the difference in switching behavior for the specific *t*_top,nom_ = 6 nm sample to the smaller AFM layer thickness deduced via XRR.

Overall, hysteresis curves clearly show that a strong IL coupling is present in our spin-valve-like heterostructures. In the following, we make use of this strong IL coupling between the two separated FM layers to demonstrate the MI control of the entire heterostructure, including the EB layers, by an electrochemical thickness modification of the top layer only.

### Control of Exchange Bias by the Electrochemical Reduction of the Top Layer

For our MI study, we place our spin-valve-like heterostructures in an electrochemical cell filled with an alkaline electrolyte, which is designed for the use in a MOKE microscope. The heterostructures are then contacted as the working electrode in a three-electrode setup, with Pt wires serving as counter and reference electrodes, respectively. In this setup, MOKE hysteresis loops can then be measured *in situ* upon application of a potential against the reference electrode. We applied increasingly more negative (cathodic) potentials to the sample in a stepwise manner starting at –0.9 V. At each step, potentials are held until a stable hysteresis curve is recorded, which typically lasts less than 100 s. At a certain potential, the reduction of the FeO_*x*_ surface layer to Fe ensues on the top layer as sketched in Figure 2a and a strong modification of the hysteresis loops is observed. The change of magnetic properties during our stepwise reduction procedure is exemplified in Figure 2b for the sample with *t*_top,nom_ = 3 nm. A decrease of *H*_EB_ and *H*_c_ sets in at −1.10 V, while the maximum MI modification is reached at −1.15 V for this sample. For samples with thicker *t*_top,nom_, the behavior of *H*_EB_ and *H*_c_ with decreasing potentials is similar, while the maximum MI modification is already attained at −1.10 V (not shown). The potential necessary for the strong MI effect agrees well with previous findings on EB Fe thin films,^33^ where a modification of *H*_EB_ was observed at −1.09 V vs Pt. Cyclic voltammograms (shown in Figure S2 in the Supporting Information) further demonstrate that the overall electrochemical behavior of our samples is analogous to Fe thin films in alkaline solution, indicating that only the top Fe is electrochemically active in our heterostructures. In the following, we show hysteresis loops at a fixed potential corresponding to the strongest MI effect.

**Figure 2:**
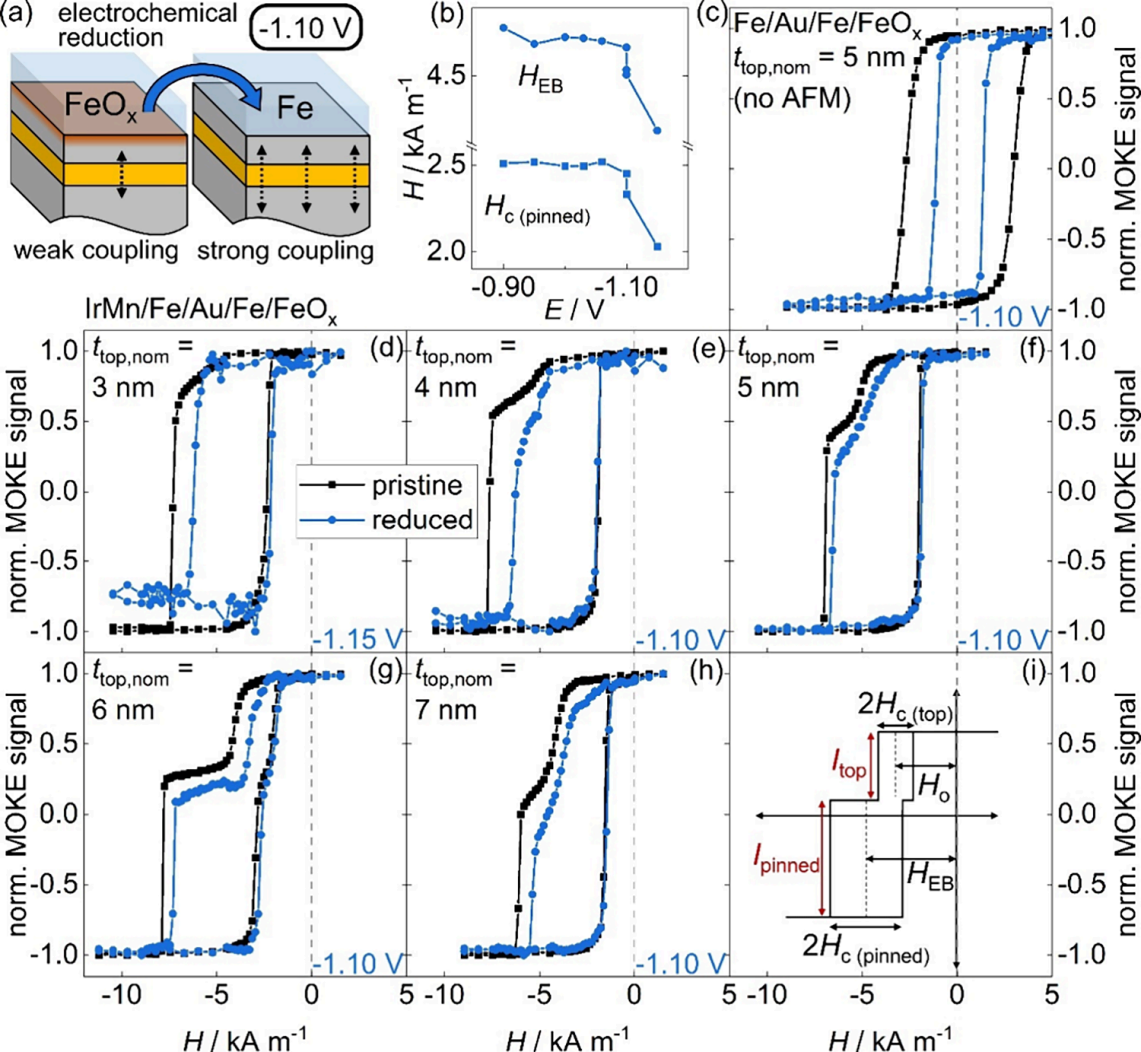
Magneto-ionic control of coupled spin-valve-like heterostructures. (a) Schematic of the MI mechanism, which is based on the increase in top Fe layer thickness upon electrochemical reduction and a concomitant strengthening of the IL coupling of both Fe layers. (b) Exchange bias field *H*_EB_ and coercivity *H*_c (pinned)_ as a function of potential *E* for the sample with t_top,nom_ = 3 nm. (c–h) Hysteresis loops in the pristine (black, squares) and reduced (blue, circles) states. (c) A Fe/Au/Fe/FeO_*x*_ reference sample without an AFM layer is shown for comparison, while (d–h) shows IrMn/Fe/Au/Fe/FeO_*x*_ samples with increasing nominal top Fe layer thickness *t*_top,nom_. The true top Fe layer thickness is smaller due to the native FeO_*x*_ surface layer on our samples. Reduction potentials are stated in the respective graphs. All pristine and reduced hysteresis loops are normalized independently. (i) Characteristic fields in a spin-valve hysteresis loop.

In Figure 2c–h, hysteresis loops in the reduced state upon application of a potential are shown for our heterostructures in blue in comparison to loops in the pristine (air-exposed) states in black. The applied potential is given in each plot. We start discussing hysteresis loops for a Fe­(10 nm)/Au­(4 nm)/Fe­(5 nm) heterostructure (*t*_top,nom_ = 5 nm) without an AFM underlayer in Figure 2c, which is shown as a reference in comparison to the EB spin-valve-like heterostructures. In the absence of the AFM underlayer, loops are centered around zero field (dashed line) in both the pristine state (black, squares) and the reduced state (blue, circles). Furthermore, loops in both states display single-step switching behavior, which indicates a strong magnetic coupling of the two Fe layers through the Au IL also in this structure, which allows collective magnetization reversal. Coercivity decreases from the pristine to the reduced state, while a single-step hysteresis loop is retained. Such a decrease of coercivity is consistent with MI studies on single-layer Fe thin films, where the same trend was observed upon reduction of the native FeO_*x*_ layer, which was mainly attributed to the removal of pinning sites for domain walls at the Fe/FeO_*x*_ interface.^46,51^ In our Fe/Au/Fe structure, only the top Fe layer is both covered by a native FeO_*x*_ surface layer and in contact with the electrolyte, which locally constrains the reduction reaction to the top Fe layer. Therefore, only the top Fe layer is the active MI layer in our heterostructures. Remarkably, however, no separate loop with a decreased coercivity arises for the active MI top layer upon reduction, but the whole single-step hysteresis loop changes coercivity instead. As this single-step loop is the result of the collective switching of both Fe layers, the magnetic properties of both layers can be controlled by the MI reduction treatment of the top layer, as a result of the strong ferromagnetic IL coupling in our heterostructures.

This separation of an MI active top layer and a magnetically controllable, but chemically unaffected lower layer can be exploited for the voltage control of EB in spin-valve-like heterostructures. For the discussion of spin-valve-like hysteresis loops in the following, it is convenient to define characteristic fields^50^ of such a loop, which are schematically sketched in Figure 2i. The EB field and coercivity of the pinned layer are denoted as *H*_EB_ and *H*_c(pinned)_, while the offset field and the coercivity of the top layer are denoted as *H*_o_ and *H*_c(top)_, respectively, in the following. The contributions of both layers to a double-step loop are indicated via the intensities *I*_top_ and *I*_pinned_, which are proportional to the thicknesses of the top and pinned Fe layers, respectively.

Hysteresis loops for IrMn/Fe/Au/Fe­(*t*_top,nom_) EB spin-valve-like heterostructures with different nominal top layer thicknesses *t*_top,nom_ from 3 to 7 nm are shown in Figure 2d–h in the reduced state, in comparison to the corresponding pristine loops from Figure 1f. For all *t*_top,nom_, we observe a pronounced narrowing of the hysteresis loops upon electrochemical reduction of the FeO_*x*_ surface layer to metallic Fe (blue, circles). For *t*_top,nom_ = 3 nm in Figure 2d, a single-step loop persists in the reduced state, while for *t*_top,nom_ ≥4 nm in Figure 2e–h, a double-step hysteresis loop is retained and a separate loop for the top layer is observed. For *t*_top,nom_ ≥4 nm, it also becomes apparent that *I*_top_ constitutes a larger fraction of the total intensity upon reduction, which indicates a larger top Fe layer thickness in the reduced state. Such an increase of the top Fe layer thickness upon electrochemical reduction of FeO_*x*_ on the surface is also discussed below as the underlying mechanism of the observed MI effects in our work. In terms of characteristic fields, a decrease of *H*_o_ and *H*_c(top)_ upon reduction is observed for all loops with *t*_top,nom_ ≥4 nm. While a decrease of *H*_c(top)_ is an expected result that has been observed for single-layer Fe thin films upon reduction of a native FeO_*x*_ surface layer,^46,51^ characteristic fields for the pinned layer *H*_EB_ and *H*_c(pinned)_ also decrease for all heterostructures. Although the pinned Fe layer is isolated from the active MI top Fe layer, its properties can be controlled by the electrochemical modification of the top layer. We attribute this modification of pinned layer properties upon MI treatment of the top layer to the strong ferromagnetic IL coupling in our heterostructures.

### Modeling the Thickness Dependence of Magnetic Properties

In a previous publication,^33^ where the MI control of EB was demonstrated for the IrMn/Fe/FeO_*x*_ system with a limited reversibility, the MI effect was attributed to an increase of the Fe layer thickness upon reduction of the native FeO_*x*_ surface layer. This assumes that the FeO_*x*_ layer has a vanishing magnetization compared to Fe, and the only change in magnetization stems from the variation in top Fe layer thickness. In order to assess whether such a change in top Fe layer thickness *t*_top_ can account for the MI effect in the present IrMn/Fe/Au/Fe/FeO_*x*_ heterostructures, we performed numerical hysteresis loop calculations for different *t*_top_ based on a modified Stoner–Wohlfarth (SW) model. In the SW model, both FM layers are treated as single-domain layers with a uniform (in-plane) magnetization in which magnetization reverses via coherent rotation. Although the formation and propagation of magnetic domains is not captured by this simple model, it can still provide valuable insight into magnetization reversal. In both EB bilayers and spin-valve heterostructures, extended SW models have been used to successfully model their magnetic behavior.^3,49,50,52^

For our model, we define conventions for the magnetic energy terms in Figure 3a, which allows us to write the following expression for the total energy per unit area *E*/*A:* 
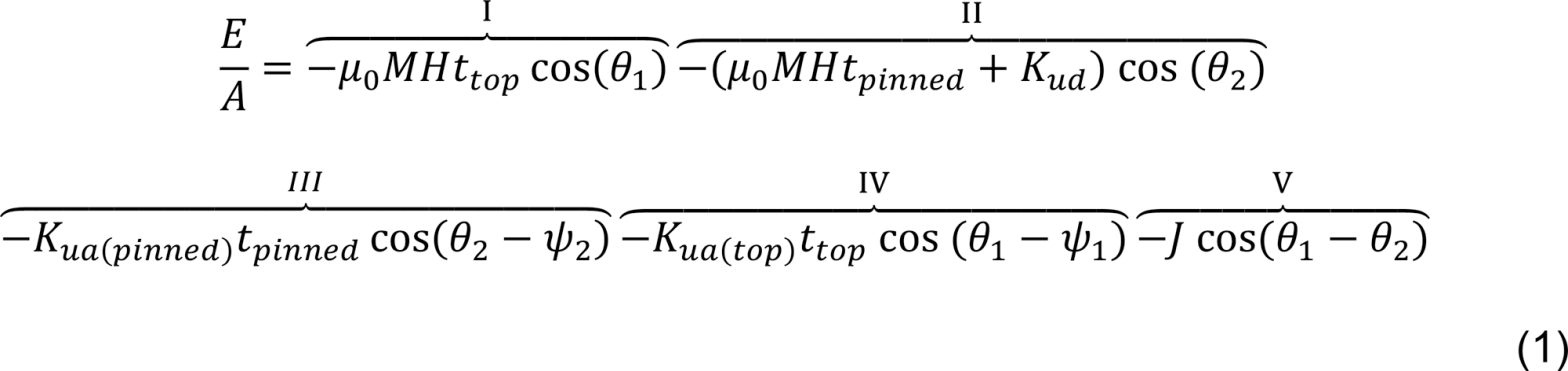
where μ_0_ is the vacuum permeability, *M* is the saturation magnetization of iron (1.71 × 10^6^ A m^–1^),^53^ *H* is the applied external field, *t*_top_ and *t*_pinned_ are the thicknesses of the top and pinned FM layers, respectively, *K*_ua(top)_ and *K*_ua(pinned)_ are the uniaxial anisotropy constants of the top and pinned FM layers, *K*_ud_ is the unidirectional (EB) anisotropy constant, and *J* is the IL coupling constant. The direction of the unidirectional anisotropy is equal to the applied field direction, as we calculate hysteresis loops only for the nominal easy axis direction in which all measurements are performed. The angles θ_1_ and θ_2_ are defined between magnetization directions of the top and pinned FM layers and the magnetic field direction, respectively. As sample deposition in an external magnetic field induces a uniaxial anisotropy in FM layers,^54^ intrinsic uniaxial anisotropy contributions are assumed in our model for both layers, with the angles ψ_1_ and ψ_2_ being the directions of the uniaxial anisotropies of top and pinned FM layers with respect to the magnetic field direction. Energy terms in this expression in Equation 1 account for magnetostatic (Zeeman) energies (terms I+II), anisotropy energies of both FM layers (terms III+IV), unidirectional EB anisotropy (included in term II), and IL coupling (term V).

**Figure 3:**
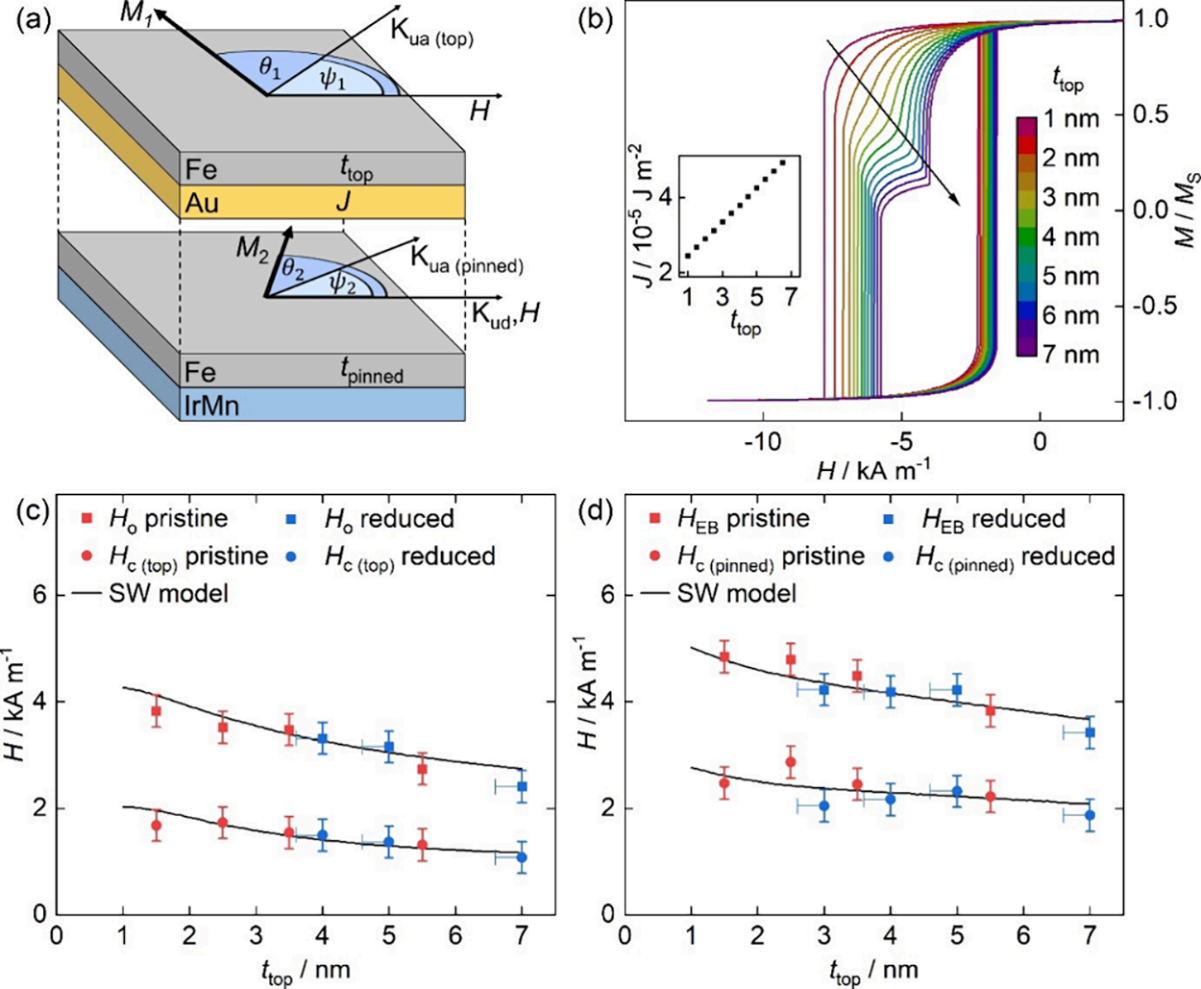
Modeled hysteresis loops for IrMn/Fe/Au/Fe heterostructures and derived characteristic fields as a function of *t*_top_ in comparison to experimental values. (a) Geometric representation of the parameters used in the extended SW model. (b) Modeled hysteresis loops for different top-layer Fe thickness *t*_top_, which is the only free parameter in the model. The IL coupling strength *J* is set to vary with *t*_top_, as shown in the inset. The arrow indicates the trend in hysteresis shape with increasing *t*_top_. (c, d) A comparison of the characteristic fields extracted from experiments in Figure 2 in the pristine and reduced states and the ones extracted from the SW model is shown for (c) the top layer and (d) the pinned layer. Note that the experimental fields for sample with the thinner IrMn AFM layer (*t*_top,nom_ = 6 nm) have been excluded from this comparison.

In the Supporting Information (S3), we show that for this total energy, analytical expressions for the top Fe layer thickness dependence of *H*_EB_ can also be derived for two limiting cases of the model, i.e., no coupling and infinitely strong coupling between the FM layers. The strong coupling limit can already describe variations of *H*_EB_ in our sample series with different top layer thicknesses reasonably well, as seen in Figure S3 in the Supporting Information. For an accurate description of all characteristic fields in our spin-valve-like structure as a function of thickness, however, we need to consider a varying coupling strength *J* as a function of top layer thickness *t*_top_ and calculate hysteresis loops in the SW model numerically. To find such an expression for *J*, we discuss the origin of IL coupling in our heterostructure in the following.

The IL coupling in Fe/Au/Fe trilayers, which are the interacting layers in our heterostructure, has been intensively investigated in the past.^55–57^ Two main contributions to IL coupling could be responsible for a net ferromagnetic interaction between the two Fe layers, which we always observe in our heterostructures. These contributions are (i) an oscillating RKKY-type (Ruderman–Kittel–Kasuya–Yosida) coupling and (ii) a Néel-type (or orange peel) coupling caused by rough interfaces. A contribution of direct exchange coupling through pinholes in the Au layer is considered unlikely, as Au layers appear microscopically closed both in TEM images in Figure 1d and EDS maps in Figure S1 in the Supporting Information for representative cross sections of our heterostructures.

For smooth Fe/Au/Fe trilayers, an RKKY-type coupling has previously been reported,^55,56^ where the IL coupling constant *J* oscillates between ferromagnetic and antiferromagnetic coupling as a function of Au IL thickness.

In our coupled spin-valve-like heterostructure, we used Au IL thicknesses in the range <6 nm, where the IL coupling was found to be ferromagnetic.^55^ The Fe/Au/Fe­(5 nm) sample without the IrMn AFM layer, which we have sputtered as a reference, also showed ferromagnetic coupling at room temperature in Figure 2c. In our heterostructures, we have not found any signs of an antiferromagnetic IL coupling.

In our case, however, there might be a stronger ferromagnetic contribution to the IL coupling constant *J* stemming from interface roughness.^58,59^ Stray fields of two FM surfaces with correlated roughness or waviness can cause a long-range ferromagnetic interaction between the two layers, which is also known as Néel-type coupling or orange peel coupling. Our heterostructures grow in columnar grains during sputtering, as apparent from the TEM image in Figure 1d. The grain structure is defined by the Au buffer layer and subsequently transferred to the upper layers. Such a grain structure is associated with a degree of correlated waviness that causes Néel coupling.^59,60^ The root-mean-square (RMS) roughness of our untreated heterostructures as deduced from atomic force microscopy is between 0.2 and 0.35 nm with no large-scale waviness apparent (see Figure S4 in the Supporting Information). Therefore, it is reasonable to assume that the columnar grain structure is responsible for the only prominent form of waviness in our samples. In the Supporting Information, Figure S5, we demonstrate that coupling constants for a Néel-type coupling derived from structural parameters only (grain size and RMS roughness) reproduce the coupling constants needed for the accurate modeling our hysteresis curves reasonably well. We therefore think that Néel coupling is the dominant form of interlayer coupling in our samples.

A biquadratic coupling contribution, favoring a 90° alignment of the magnetization in the two Fe layers, has also been discussed in Fe/Au/Fe trilayers^56^ and spin-valve structures before. However, for simplicity, we did not explicitly include such an interaction in our extended SW model, as it can also manifest itself indirectly in the misalignment angle ψ_1_ (see S6 in the Supporting Information). A size distribution of AFM grains has also been considered in previous extended SW models for EB for the description of time-dependent magnetic properties,^43,49,52^ which we also neglect in this simple model. For our SW model, we take into account a linear increase of *J* with top layer thickness *t*_top_, which is expected for the Néel interaction in the limit of thin top layers,^61^ as well as an additional constant ferromagnetic contribution accounting for a ferromagnetic coupling independent of roughness. The function we used for *J* is shown in the inset of Figure 3b, which can be written as *J* = 0.02 mJ m^–2^ + 0.0045 mJ m^–2^ nm^–1^ × *t*_top_, with *t*_top_ given in nm. Note that for the modeling of our coupled heterostructures, *J* ranges up to 0.05 mJ m^–2^, which is slightly larger as compared to typical spin-valve structures.^3,62^ This stronger coupling is caused by the use of Fe instead of magnetically softer materials,^3,62^ which allows a more rigid coupling.

We obtain modeled hysteresis loops as a function of *t*_top_ in Figure 3b when we use *J* as above, as well as the following set of parameters selected to fit experimental hysteresis loops: *t*_pinned_ = 10 nm, ψ_1_ = 30°, ψ_2_ = 15°, *K*_ua(pinned)_ = 5.4 kJ m^–3^, *K*_ua(top)_ = 2.5 kJ m^–3^, and *K*_ud_ = 1.2 × 10^–4^ J m^–2^. A significant misalignment between uniaxial and unidirectional anisotropy directions is known in EB bilayers with small *K*_ua_/*K*_ud_ (20° for NiFe/IrMn^63^), which is caused by magnetic frustration at imperfect (rough) interfaces.^63^ The comparably large misalignment of the uniaxial anisotropy axis of the top layer with the magnetic field axis (ψ_1_= 30°) might be a hint that a biquadratic contribution to the IL coupling is present in our heterostructure (compare S6 in the Supporting Information). Note that all parameters besides top layer thickness *t*_top_ and coupling strength *J* (which is a function of *t*_top_) are fixed for the calculation of hysteresis loops.

In general, modeled hysteresis loops for different top layer thicknesses can reproduce the behavior of the experimental curves for different *t*_top_ in Figure 1f accurately. To facilitate direct comparison, we extracted the characteristic fields from experimental loops in pristine and reduced states in Figure 2d–h, as well as from our modeled hysteresis loops. Fields *H*_o_ and *H*_c(top)_ for the top layer are shown as a function of *t*_top_ in Figure 3c, while fields *H*_EB_ and *H*_c(pinned)_ for the pinned layer are given in Figure 3d. As we cannot monitor the thickness of the Fe layer *in situ*, we set *t*_top_ to the nominal sputtered Fe thickness *t*_top,nom_ for the reduced state (blue), assuming a full reduction of the FeO_*x*_ surface layer. Error bars on the *x*-axis account for a possibly thinner Fe layer in case only a partial reduction occurs. In the pristine state (red), *t*_top_ is the nominal thickness *t*_top,nom_ reduced by 1.5 nm, which was found to be the thickness of an Fe layer that undergoes oxidation.^48^ Values extracted from our modeled hysteresis loops are plotted as a black line.

The comparison shows that our extended SW model fully captures the trends in the characteristic fields of our experimental curves in Figure 3c,d. We obtain a quantitative agreement of modeled fields with experimental fields for both pristine and reduced states for most thicknesses *t*_top_. The experimental sample with a nominal top layer thickness of *t*_top,nom_ = 6 nm, where a much thinner AFM layer than nominally expected was found, has been excluded from this comparison of model and experiment. In the Supporting Information Figure S7, however, we show that the hysteresis loops for this sample can still be modeled reasonably well in our SW model by adapting the unidirectional anisotropy constant accounting for a stronger EB and weaker IL coupling.

Modeling hysteresis not only allows understanding the experimental hysteresis loops as a function of *t*_top_ in the pristine state, but it can also be used to interpret the MI effect in our spin-valve-like heterostructure. By assuming an increase in top Fe layer thickness by 1.5 nm upon application of a reduction potential, which corresponds to a full electrochemical reduction of FeO_*x*_, our extended SW model adequately describes changes in hysteresis loops and characteristic fields in our EB IrMn/Fe/Au/Fe/FeO_*x*_ spin-valve-like structure upon application of a reduction potential for all nominal top layer thicknesses in Figure 3c,d. An increase in top Fe layer thickness and the concomitant strengthening of the IL coupling, as sketched in Figure 2a, can thus explain the observed MI-induced hysteresis loop changes, in agreement with previous works.^33^

### Reversible Switching between Single- and Double-Step Hysteresis Loops

Lastly, we seek to exploit our MI effect to demonstrate the reversible control of EB and an ON- and OFF-switching functionality of a spin-valve-like hysteresis loop. For that purpose, we use a specific heterostructure Au­(5 nm)/IrMn­(30 nm)/Fe­(10 nm)/ Au­(5 nm)/Fe­(6 nm), which has a slightly thicker Au spacer layer compared to the ones before, resulting in a weaker coupling between both FM layers. The hysteresis loop in the pristine state resembles a classical EB loop (see Figure S8 in the Supporting Information), with only a minor noticeable contribution from the top layer.

An initial stepwise electrochemical reduction procedure for this sample indicated the occurrence of a double-step loop at sufficiently negative reduction voltages (see Figure S9in the Supporting Information). We build upon this in Figure 4, where we demonstrate the reversible toggling between double-step (ON) and single-step loop (OFF) behavior upon electrochemical reduction and subsequent oxidation steps. Figure 4a shows hysteresis loops in the oxidized state (red) along with MOKE microscopy images at certain points of the loop, while Figure 4b depicts the same for the corresponding reduced state (blue). Applied potentials for this experiment and derived characteristic fields are plotted in Figure 4c using the same color coding (values upon oxidation as red squares, values upon reduction as blue circles). Hysteresis loops are recorded over 40 measurement steps (20 reduction steps at −1.15 V/20 oxidation steps at −0.02 V). The loop associated with the top Fe layer appears only upon application of the reduction potential in (b) and vanishes again upon oxidation in (a), which allows an extraction of characteristic fields *H*_o_ and *H*_c(top)_ only in the reduced state. In Figure S10 in the Supporting Information, we demonstrate that double-step loops in the reduced state also return to single-step loops without the application of an external potential, i.e., via spontaneous oxidation under open circuit conditions. Besides the toggling between single-step and double-step loops, EB of the pinned layer is also reversibly modified by the same electrochemical treatment. In the reduced states, *H*_EB_ is about 0.4 kA m^–1^ smaller compared to the oxidized state, while also a decrease in *H*_c_ of similar magnitude is observed.

**Figure 4:**
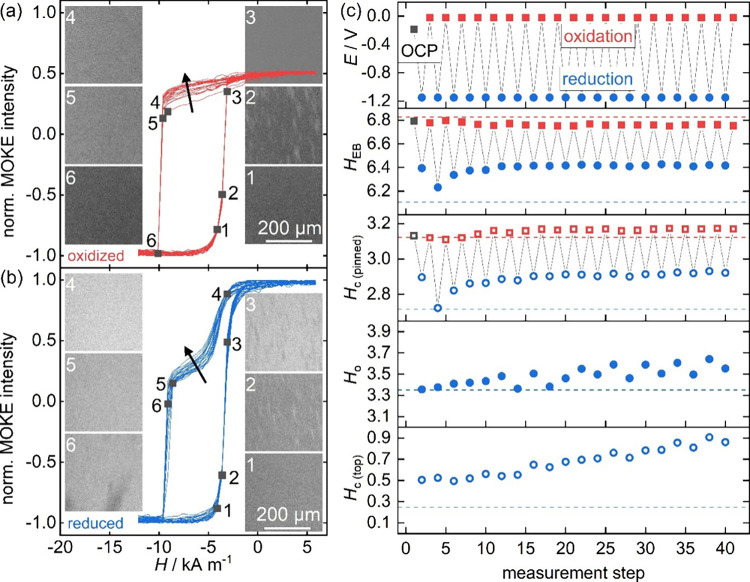
MI switching between single- and double-step hysteresis loops in a Au­(5 nm)/IrMn­(30 nm)/Fe­(10 nm)/Au­(5 nm)/Fe­(6 nm) heterostructure. Loops in the (a) oxidized state in red and (b) reduced state in blue are shown over 40 measurements (20 in each state). Black arrows indicate the trend with increasing number of switching steps. All loops are normalized using the saturation intensity in the reduced state. Corresponding domain images recorded during the first oxidation/reduction loops are shown as insets in (a) and (b), with the numbers indicating the position in the loop. (c) Applied potential *E* and the characteristic fields extracted from the loops over all 40 measurement steps (values upon oxidation as red squares, values upon reduction as blue circles). Dashed horizontal lines represent the fields extracted from modeled loops, parameters are given in the Supporting Information Figure S11. All fields are in kA m^–1^.

Domain images in both oxidized and reduced states in Figure 4a and Figure 4b, respectively, show that the magnetization reversal mechanism is similar in both cases. While in the field sweep to positive fields (1 to 3) magnetization reverses via the nucleation of many vertically elongated domains with an average width of ∼20 μm, in the field sweep to negative fields (4 to 6), the reversal mechanism is a more rapid domain formation and domain-wall motion, which is only captured in the MOKE image 6 in Figure 4b. Such an asymmetric magnetization reversal in positive and negative sweep directions has previously been found in EB layers.^64^ No domains could be observed during reversal of the top layer loop in Figure 4b, which could be an indication that the easy axis of the top layer does not coincide with the EB anisotropy direction, although we have not conducted an angle-dependent *in situ* study to confirm this. The similar reversal mechanism in both oxidized and reduced states indicates that our MI effect is not predominantly caused by changes in anisotropy or modified pinning sites on the surface,^46^ which would likely affect the magnetic domain structure upon magnetization reversal. In line with our results in the previous section, we instead suggest that the overall micromagnetic energy landscape is changed electrochemically upon voltage application. We therefore continue our discussion of the MI ON and OFF switching in this spin-valve-like structure using our extended SW model and interpret the observed effect again via a thickness modulation of the top Fe layer in the following.

Despite the reversal mechanism via domain formation, loops in both oxidized and reduced state can again be described using our SW model (see Figure S11 in the Supporting Information). Magnetic properties from the SW model have been found to come close to experimental values when the magnetization reversal is dominated by domain nucleation.^65^ This could also be the case for our coupled spin valves judging from the domain images in Figure 4a,b. For the modeling of our hysteresis loops, we had to use a larger misalignment angle of the uniaxial anisotropy for the top layer (ψ_1_= 60°) compared to the loops in Figure 3b. We hypothesize that this is caused by a stronger biquadratic coupling contribution compared to the heterostructures with a thinner Au IL, in agreement with literature findings of a more dominant biquadratic coupling in Fe/metal/Fe sandwich structures at larger IL metal thicknesses.^56^ Such a misaligned anisotropy axis could also explain the absence of domains during magnetization reversal of the top layer in our MOKE images measured along the EB axis, as domains magnetized off-axis would show fainter contrast for the used longitudinal MOKE sensitivity along the EB axis.

Based on the SW model, we can interpret the MI switching between single-step and double-step loops by an increase in the top Fe layer thickness. An increasing Fe layer thickness leads to an increased anisotropy energy of the top layer in the first place, but also an increase in the IL coupling energy in line with a predominant Néel-type coupling as outlined above. In short, the interplay of the lower anisotropy energy of the top layer with other energy terms suppresses hysteresis of the top layer in the oxidized state. The energy balance is shifted in favor of anisotropy energy in the reduced state, which allows hysteresis to develop. Such a switching between single- and double-step loops should allow the voltage control of giant magnetoresistance (GMR) in spin-valve-like structures (see Figure S12 in the Supporting Information), which could find application for tunable magnetic field sensors.^66^ Furthermore, MI spin-valve-like structures might allow controlling the polarization of spin currents via voltage gating in the future. The IL coupling in our structures, which is the key ingredient for MI functionalization of the pinned layer, causes an offset field *H*_o_ for the top layer loop, that is, so far, suboptimal for spin valves intended to switch around zero field. However, a magneto-ionic fine-tuning of the top layer loop around nonzero fields could be beneficial for field sensors with adjustable sensitivity via *H*_o_.

Finally, we discuss the control of EB for this spin-valve-like heterostructure in Figure 4c, which served as the original motivation for this work. The indirect control of EB through an Au IL reduced the magnitude of the MI effect compared to a previous study on the IrMn/Fe/FeO_*x*_ system^33^ from Δ*H*_EB_ ∼3 to ∼0.4 kA m^–1^ for comparable top Fe layer thicknesses (*t*_top,nom_ = 6 nm here, 7 nm in the reference) and comparably low potentials (*E* = −1.15 V vs Pt here, −1.03 V vs Pt in the reference). Despite the reduced magnitude of the MI effect, the spin-valve-like heterostructure indeed showed a drastic improvement in the reversibility of the EB modification of over 40 measurement steps here, while the EB modification was only partly reversible after reduction in our previous study. The isolation of the sensitive EB layer in a spin-valve-like structure thus allowed turning a partly reversible EB modification by MI^33^ into a reversible one.

While the toggling between single-step and double-step loops was also reversible over 40 cycles, characteristic fields for the top layer in the reduced state only, *H*_o_ and *H*_c(top)_, drift toward slightly larger values in the course of the experiment. As this layer is in direct contact with the electrolyte in our setting, we ascribe this drift to an increasing roughness upon MI cycling. Such an increased surface roughness was also confirmed in AFM images after cycling over 40 measurement steps (see Figure S4 in the Supporting Information). Characteristic fields for the pinned layer, *H*_EB_ and *H*_c(pinned)_, are constant for both oxidized and reduced states over 40 measurement steps, with only small variations within the uncertainty of the field measurement (Δ*H* = 0.25 kA m^–1^). Remarkably, the MI modification of EB seems to be largely unaffected by these changes on the surface layer, indicating that the separation of the EB layer from the top surface makes our MI effect not only reversible but also robust with respect to changes on the surface layer.

While the MI control of IL coupling has previously been demonstrated based on the RKKY interaction,^37,67^ our work focuses on the MI control of a protected layer that is mediated by IL coupling, rather than controlling the interaction itself. This approach should allow controlling the protected layer irrespective of the sign of the IL coupling, as outlined in Figure S13 in the Supporting Information. Future MI devices could benefit from this proposed mechanism in multiple ways: (I) The isolation of sensitive magnetic interfaces from the active MI layer, i.e., the layer affected by electrochemical reactions, by an “interaction layer” could present a general strategy for improving reversibility in MI devices. (II) As the MI effect for the protected layer(s) is exclusively mediated by the magnetic IL coupling, our approach can be easily transferred to other active MI top layers that are based on different ionic species. (III) The limited penetration depth of larger ionic species, which often restricts MI effects to the surface, can be overcome by a magnetic coupling to lower layers. (IV) The limited switching speed of current MI devices could also be improved in our design, as ions only have to influence a small fraction of the whole structure for an MI effect over the total volume.

## Conclusions

In this work, we have shown that EB in IrMn/Fe/Au/Fe/FeO_*x*_ coupled spin-valve-like heterostructures can be controlled by an electrochemical modification of the top Fe layer at low voltage (∼1 V). We found that in our heterostructures, not only the hysteresis loop of the active MI top layer can be controlled upon electrochemical reduction, but also the loop associated with the separated EB layers is significantly changed upon MI treatment. By studying the magnetic and MI behavior of our spin-valve-like heterostructures as a function of top Fe layer thickness, we found a strong ferromagnetic coupling of both Fe layers to be responsible for the MI modulation of EB through the Au IL.

In analogy to a previous study on the IrMn/Fe/FeO_*x*_ EB system,^33^ we proposed that the main underlying mechanism for our MI effect is the voltage-induced electrochemical reduction of a native FeO_*x*_ surface layer into a metallic Fe layer upon application of reduction potentials and a concomitant thickness increase of the top FM layer. By assuming a full reduction of the FeO_*x*_ surface layer on the one hand, and a reduced thickness of the Fe layer by 1.5 nm in the pristine state accounting for the oxide layer, we could successfully model hysteresis loops in pristine and reduced states in an extended SW model. A single set of parameters was sufficient to reproduce our experimental data in both pristine and reduced states, with the top layer thickness as the only free parameter.

Furthermore, we could also demonstrate the reversible toggling between single-step and double-step hysteresis loops in an IL-coupled spin-valve-like structure with a slightly thicker Au IL. The hysteresis loop for the top Fe layer emerges only upon application of a reduction potential and vanishes again upon application of an oxidation potential. Such an ON/OFF spin-valve functionality should allow controlling magnetoresistance in GMR stacks and could pave the way for magnetic field sensors with adjustable sensitivity. At the same time, a reversible decrease and increase of the EB field is observed in the pinned layer, with a drastic improvement in reversibility of the MI effect compared to the IrMn/Fe/FeO_*x*_ EB system. This improvement in reversibility is attributed to the isolation of the sensitive EB interface from the electrochemical reaction in our spin-valve-like structure. We therefore propose the isolation of magnetically sensitive surfaces from electrochemical reactions via a coupling layer as a general strategy to obtain MI devices with advanced reversibility. Our results for the MI control of spin-valve-like structures may also serve as a starting point for the voltage control of even more complex layered magnetic systems, such as synthetic antiferromagnets^68^ or noncollinear magnetic layered structures,^69^ which have attracted attention as MRAM architectures for future magnetic data storage devices.

## Experimental Section

### Sample Preparation

Our spin-valve-like Au­(5 nm)/Ir_17_Mn_83_(30 nm)/Fe­(10 nm)/Au­(4 nm; 5 nm)/Fe­(3 nm; 4 nm; 5 nm; 6 nm; 7 nm) heterostructures were deposited on Si substrates with a native SiO_*x*_ layer and a size of 1 × 1 cm^2^ using rf-magnetron sputtering. Sputtering was conducted at room temperature at a working pressure of 10^–2^ mbar under an Ar atmosphere. To set the EB direction, an external magnetic field (28 kA m^–1^) was applied parallel to the sample plane during sputtering. Precharacterization of each sample directly after sputtering was performed via vibrating sample magnetometry (VSM) and longitudinal MOKE magnetometry (not shown).

### X-ray Reflectivity

A Rigaku SmartLab instrument equipped with a rotating 9 kW Cu anode (λ = 0.154 nm) and a Cu K_β_ filter was used for the XRR measurements. XRR curves were recorded between 0 and 10° in steps of 0.004° in parallel beam geometry. Samples were used for MI tests before XRR characterization. The resulting curves were fitted with GenX (v3.6.27)^70^ software in the spec_inhom model, without inhomogeneities being necessary to fit the curves.

### TEM Measurements

The TEM cross-section samples were fabricated by means of Thermo Fisher FIB Helios NanoLab 600i on molybdenum grids. High-resolution TEM images were acquired with the TF Titan^3^ 80–300 double aberration-corrected (scanning) transmission electron microscope operating at 300 kV accelerating voltage, which is equipped with a Gatan OneView Camera Model 1095. Calibration was confirmed by the [002] reflection of the silicon substrate.

### MOKE Microscopy/Magnetometry

A MOKE microscope (evico magnetics) was employed for magnetic and MI characterization of our samples. In-plane magnetic fields were applied parallel to the EB setting field direction, while a 20× objective lens (Zeiss LD Plan-Neofluar) with a correction collar to adjust for the cover glass thickness was used to image the magnetic surface. Magnetic fields were generated using an electromagnet, which was calibrated using a Hall probe.

### MI Experiments

A potentiostat (BioLogic SP50) was used to apply potentials in a three-electrode electrochemical cell designed for the use with the MOKE microscope. A sketch of the cell design can be found in a prior publication.^33^ Pt wires were used both as reference and counter electrodes in this setup, while the samples were connected as working electrodes. An O-ring was used to mask a defined area (0.385 cm^2^) on the sample that is in contact with the electrolyte and to seal the cell. The electrolyte compartment was filled with 200 μL of electrolyte solution and covered with a glass coverslip. MOKE images were focused on the sample surface through the electrolyte solution. For the experiments demonstrating the control of EB, we used a 1 M LiOH electrolyte solution, while for the control of spin-valve-like loops, 1 M KOH was used as the electrolyte. It has been shown that for the Fe/FeO_*x*_ systems, both of these electrolytes produce the same MI effect and the influence of the cations (Li^+^ or K^+^) is marginal.^51^ Acidic electrolytes, on the other hand, cannot be used with this system due to Fe dissolution in a large potential range.

For the reduction of the FeO_*x*_ layer in Figure 2b, potentials were decreased in steps (−0.90, −0.95, −1.00, −1.03, −1.06, −1.10, and −1.15 V) until a change in hysteresis loop was observed. For the extraction of characteristic fields of the top and pinned layer from the experimental loops, we use cuts through loops at fixed magnetization levels. As the total magnetization is the sum of magnetization of top and pinned layers, we can define the magnetization level of the individual layer loops using the thickness ratio of the two layers. Characteristic fields for the top layer are extracted at the magnetization level *M*/*M*_s_ = 1 – *t*_top_/(*t*_pinned_ + *t*_top_), while characteristic fields for the pinned layer are obtained at the magnetization level *M*/*M*_s_ = −1 + (*t*_pinned_)/(*t*_pinned_ + *t*_top_). For the thickness of the top layer in the pristine state, we assume that 1.5 nm of the Fe layer is oxidized^48^ and subtract this thickness from the nominal top layer thickness *t*_top,nom_. For the reduced states, the nominal thickness *t*_top,nom_ is used to obtain magnetization levels. Hysteresis loops and domain images are recorded with longitudinal sensitivity in the EB direction, which corresponds to the vertical direction in the images.

### SW Modeling of the Hysteresis Loops

To find angles of the magnetization directions θ_1_ and θ_2_, where the expression for the total energy in the extended SW model is minimized for a fixed set of parameters, we computed the partial derivatives with respect to the angles and solved for the zeros of these derivatives numerically using the MATLAB software for an initial magnetic field. We then model hysteresis loops by following the local angular minimum of the total energy along the steepest gradient, while varying the magnetic field *H*. *H* was varied in steps of 0.02 kA m^–1^, while the magnetization directions had an accuracy of 0.5°. The normalized longitudinal magnetization was calculated from the minimum θ angles as $\frac{t_{\mathrm{top}}\cos\left(\theta_{1}\right)+t_{\mathrm{pinned}}\cos\left(\theta_{2}\right)}{t_{\mathrm{top}}+t_{\mathrm{pinned}}}$ , where *t*_top_ and *t*_pinned_ are the thicknesses of top and pinned layer, respectively. Plotting magnetization over *H* yields modeled hysteresis loops for a given set of parameters, which are specified in the text. Hysteresis loops are modeled for thickness steps of 0.05 nm, to allow an almost continuous extraction of characteristic fields.

## Supplementary Material



## Data Availability

The data for this publication is openly available via the Zenodo repository at https://doi.org/10.5281/zenodo.16902546, reference number 16902546.

## Notes

Layer thicknesses for spin-valve heterostructures from X-ray reflectivity and transmission electron microscopy, energy-dispersive X-ray spectroscopy - elemental maps and profiles, cyclic voltammetry experiments, analytical expressions for the exchange bias field, atomic force microscopy images, estimation of coupling constants, Stoner–Wohlfarth modeled hysteresis loops, potential-dependent hysteresis loops, and calculation of the giant magnetoresistance (PDF)
