# Supplementary material for: Reversible Magneto-Ionic Control of Exchange Bias in Coupled Spin-Valve-Like Heterostructures
Source: ACS Appl Mater Interfaces. 2025 Aug 24;17(35):49671–82. doi: 10.1021/acsami.5c10187 (PMC12412114; doi:10.1021/acsami.5c10187)
Supplement: Supplementary file 1 [file am5c10187_si_001.pdf]

Supporting Information for

Reversible magneto-ionic control of exchange bias in coupled spin-valve-like heterostructures

Markus Gößler[1,], Jonas Zehner[1,2], Rico Huhnstock[3,4], Falk Röder[2,5], Rico Ehrler[6,7], Olav Hellwig[6,7,8], Arno Ehresmann[3,4], Karin Leistner[1,7]*

[1]Institute of Chemistry, Chemnitz University of Technology, 09107 Chemnitz, Germany

[2]Leibniz Institute for Solid State and Materials Research, 01069 Dresden, Germany

[3]Institute of Physics, University of Kassel, 34132 Kassel, Germany

[4]Center for Interdisciplinary Nanostructure Science and Technology (CINSaT), University of Kassel, 34132 Kassel, Germany

[5]Leibniz Institute of Polymer Research, 01069 Dresden, Germany

[6]Institute of Physics, Chemnitz University of Technology, 09107 Chemnitz, Germany

[7]Center for Materials Architectures and Integration of Nanomembranes (MAIN), Chemnitz University of Technology, 09107 Chemnitz, Germany

[8]Institute of Ion Beam Physics and Materials Research, Helmholtz-Zentrum Dresden-Rossendorf, 01328 Dresden, Germany

[*]corresponding author, e-mail: markus.goessler@chemie.tu-chemnitz.de

S1. Layer thicknesses from XRR fitting and EDS profiles

Thicknesses extracted from the SLD profiles in Figure 1 in the main text are given in Table 1. The nominal thickness of the Fe top layer is given as a reference in the first column. A nucleation Au layer with lower density below the Au seed is needed to fit the pronounced dip at around 6°. For the FeO_x layer the electron density of Fe_2O_3 is used in the fitting procedure. Thickness uncertainties stemming from the fits are generally well below 1 nm. Thicknesses of the Au interlayer, Fe top, and FeO_x layer are not clearly visible as distinct oscillations in the XRR and cannot be deconvoluted accurately for that reason. Their thicknesses are grayed out in Table 1, as values are prone to large uncertainties. The target thickness of 30 nm for the IrMn layer is significantly reduced for the samples with a top Fe layer thickness of $t_{top,nom}$ = 6 nm and $t_{top,nom}$ = 7 nm. The Software GenX (v3.6.27)[1] is used for fitting and the extraction of thicknesses.

Table 1. Individual layer thicknesses in nm obtained from fitting XRR curves of the IrMn/Fe/Au/Fe/FeO_x heterostructures with varying $t_{top,nom}$.

$t_{top,nom}$	SiO_2	Au	IrMn	Fe	Au	Fe	FeO_x (Fe_2O_3)
3	1.7	5.7	26.9	9.8	5.2	0.7	2.0
4	1.6	5.5	27.3	9.7	4.1	1.5	5.1
5	1.5	5.5	27.3	9.7	5.8	1.2	3.8
6	1.7	5.1	17.0	8.9	6.0	5.1	2.9
7	1.5	5.5	22.0	9.9	6.0	0.4	5.7

Table 2. Individual layer thicknesses in nm obtained from EDS elemental profiles of the IrMn/Fe/Au/Fe/FeO_x heterostructures with $t_{top,nom}$ = 6 nm and 7 nm.

$t_{top,nom}$	SiO_2	Au	IrMn	Fe	Au	Fe + FeO_x
6	-	5.6	16.6	8.6	5.2	8.0
7	-	5.7	22.8	8.9	5.3	8.5
uncertainty	-	0.3	0.5	0.5	0.6	0.7

For the elemental mapping and EDS profiles, High-angle annular dark field scanning TEM (HAADF-STEM) and STEM-EDS measurements were conducted using the Talos 200i operating at 200 kV acceleration voltage using a convergence semi-angle of 10.5mrad and a HAADF collection angle range of 32-192 mrad. The EDS measurements were conducted by the Thermo Fischer Dual-X detector and a low-background double-tilt specimen holder. Each sample position was tilted edge-on. Elemental mapping was performed in a region of (91.9 x 96.7) nm^2 sampled by 77 x 81 scanning points at dwell times of 25 μs and by summing over 256 drift- corrected frames. The elemental maps and profiles shown in Figure S1 correspond to the background-corrected intensities of the following absorption edges: oxygen, iron and manganese: K_α-edges, iridium and gold L_α-edges. The represented profiles are averaged in parallel direction to the layers. A spectral overlap of Ga-K edge and Ir-L_α edge can cause artifacts in the Ir-map because of Ga residuals from sample preparation. The fabrication of TEM cross section is described in the main manuscript. Thicknesses extracted from the element profiles are given in Table 2, with thickness uncertainties given in the last row.

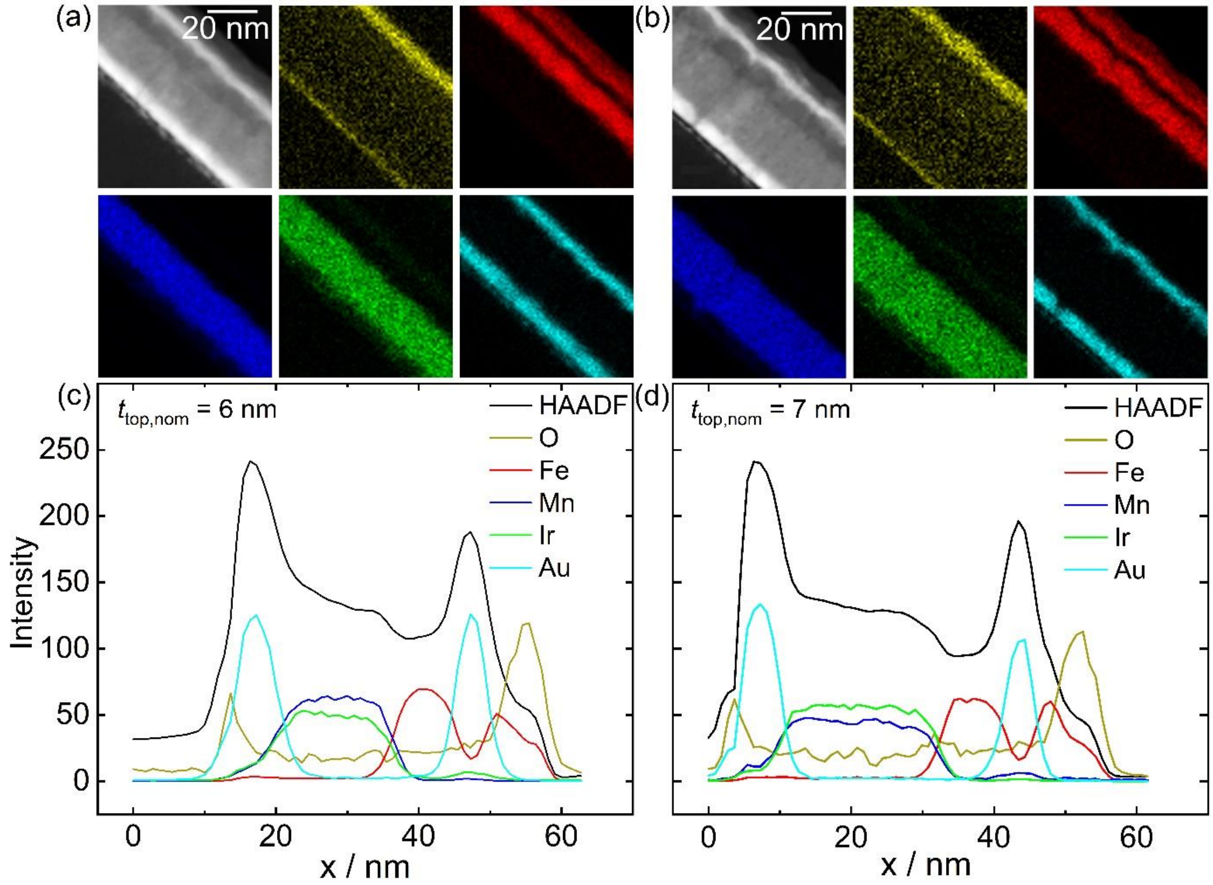

Figure S1. High-angle annular dark field (HAADF) TEM image and corresponding EDS elemental maps for samples with $t_{top,nom}$ = 6 nm (a) and $t_{top,nom}$ = 7 nm (b). The corresponding EDS profiles as a function of distance are shown in (c) and (d). The color-coding is the same for maps and profiles: yellow-oxygen, red-iron, blue-manganese, green-iridium, and cyan-gold.

S2. Cyclic voltammetry

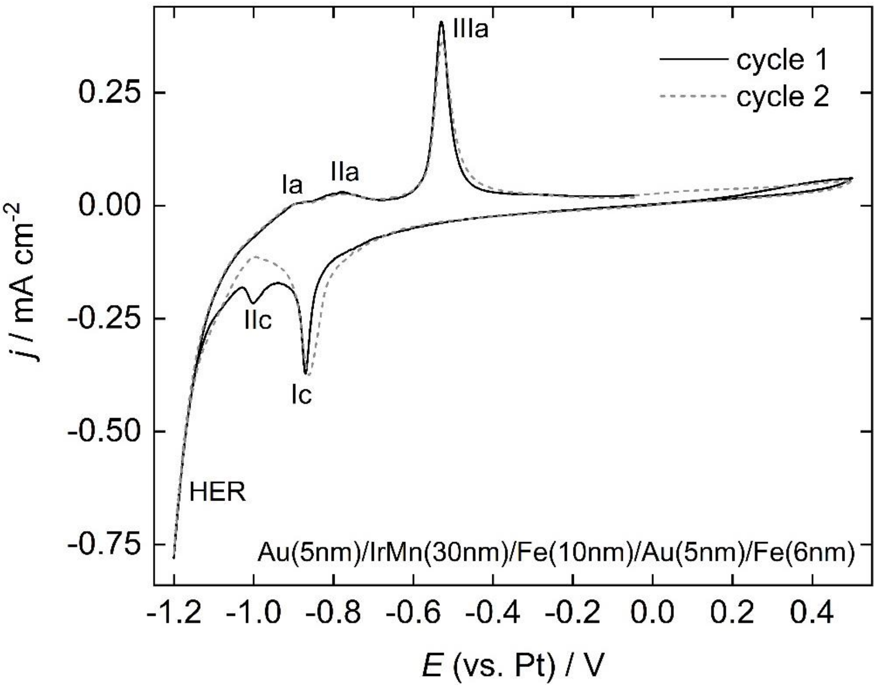

Figure S2. Cyclic voltammogram (CV) of a IrMn/Fe/Au/Fe sample ($t_{top,nom}$ = 6 nm, t_{Au} = 5 nm) recorded with a scan rate of 10 mV s^{-1} in 1 M KOH electrolyte. Two consecutive cycles are shown. This CV has been recorded after the 40 measurement steps on the same sample shown in Figure 4 in the main text.

A cyclic voltammogram (CV) recorded on a coupled spin-valve heterostructure in 1M KOH is shown in Figure S2. This CV shows analogous features compared to CVs recorded for Fe thin films in alkaline solution,[2,3] indicating that only the top Fe layer is treated electrochemically in our heterostructure The following peak assignment is based on Reference 3: peak Ia corresponds to the oxidation of adsorbed hydrogen, peak IIa is attributed to the oxidation of Fe to Fe^{2+} species, and peak IIIa is the oxidation of $Fe(OH)_2$ to Fe^{3+} species. In the following passive region Fe_3O_4 and hydrated Fe_2O_3 are expected to form. In the cathodic scan, peak Ic corresponds to the reduction of iron oxides and oxyhydroxides to $Fe(OH)_2$, while peak IIc can be attributed to the reduction of $Fe(OH)_2$ to metallic Fe. The strong current increase at strongly negative potentials is caused by the hydrogen evolution reaction (HER), which causes the formation of H_2 bubbles on our samples that can obstruct the recording of hysteresis loops using the *in situ* MOKE microscopy method. For the electrochemical switching in the main text, we used potential of -1.10 V or -1.15 V for the reduction step and a potential of -0.02 V for the oxidation step.

S3. Analytical expressions for the EB field

The general expression for the total energy in our extended SW approach reads:

$$\begin{aligned} E_{\text{tot}} = &-\mu_0 M H t_1 \cos(\theta_1) - \mu_0 M H t_2 \cos(\theta_2) - K_{\text{ud}} \cos(\theta_2 - \phi) - J \cos(\theta_1 - \theta_2) \\ &- K_{\text{ua1}}\, t_1 \cos^2(\theta_1 - \psi_1) - K_{\text{ua2}}\, t_2 \cos^2(\theta_2 - \psi_2) \end{aligned} \tag{S1}$$

For the definition of all parameters and angles the reader is referred to Fig. 3 and its description in the main manuscript. For the sake of a shorter notation, we used t_1 and t_2 instead of the thicknesses t_{top} and t_{pinned} in the main manuscript. In the following considerations, we limit ourselves in this ananlytical treatment to aligned anisotropy axes with both the EB direction and field direction in the easy axis, i.e. $\phi = 0$, $\psi_1 = 0$, $\psi_2 = 0$, which simplifies the energy expression to:

$$\begin{aligned} E_{\text{tot}} = &-\mu_0 M H t_1 \cos(\theta_1) - (\mu_0 M H t_2 + K_{\text{ud}}) \cos(\theta_2) - J \cos(\theta_1 - \theta_2) \\ &- K_{\text{ua1}}\, t_1 \cos^2(\theta_1) - K_{\text{ua2}}\, t_2 \cos^2(\theta_2) \end{aligned} \tag{S2}$$

In the Stoner-Wohlfarth model the conditions for energy minima are given by the first derivatives of total energy E_{tot} with respect to the alignment angles of magnetization θ_1 and θ_2.

$$\begin{aligned} \frac{\partial E_{\text{tot}}}{\partial \theta_1} &= \mu_0 M H t_1 \sin(\theta_1) + J \sin(\theta_1 - \theta_2) + 2K_{\text{ua1}}\, t_1 \cos(\theta_1)\sin(\theta_1) \\ \frac{\partial E_{\text{tot}}}{\partial \theta_2} &= (\mu_0 M H t_2 + K_{\text{ud}}) \sin(\theta_2) - J \sin(\theta_1 - \theta_2) + 2K_{\text{ua2}}\, t_2 \cos(\theta_2)\sin(\theta_2) \end{aligned} \tag{S3}$$

We know consider two limiting cases:

(I) strong coupling, i.e. perfect alignment of both layers, which involves $\theta_1 = \theta_2 = \theta$ for all fields H

(II) full decoupling, i.e. fully independent switching behaviour of both layers and $J = 0$.

For case (I) we get the following first partial derivatives of E_{tot}:

$$\begin{aligned}\frac{\partial E_{\mathrm{tot}}}{\partial \theta_1} &= \mu_0 M H t_1 \sin(\theta) + 2K_{\mathrm{ua1}}\, t_1 \cos(\theta)\sin(\theta) = 0 \\ \frac{\partial E_{\mathrm{tot}}}{\partial \theta_2} &= (\mu_0 M H t_2 + K_{\mathrm{ud}}) \sin(\theta) + 2K_{\mathrm{ua2}}\, t_2 \cos(\theta)\sin(\theta) = 0\end{aligned} \tag{S4}$$

Energy minima have to fulfill the following conditions:

$$\begin{aligned}\frac{\partial^2 E_{\mathrm{tot}}}{\partial \theta^2} &= \mu_0 M H t_1 \cos(\theta) + 2K_{\mathrm{ua1}}\, t_1 \cos^2(\theta) - 2K_{\mathrm{ua1}}\, t_1 \sin^2(\theta) > 0 \\ \frac{\partial^2 E_{\mathrm{tot}}}{\partial \theta^2} &= (\mu_0 M H t_2 + K_{\mathrm{ud}}) \cos(\theta) + 2K_{\mathrm{ua2}}\, t_2 \cos^2(\theta) - 2K_{\mathrm{ua2}}\, t_2 \sin^2(\theta) > 0\end{aligned} \tag{S5}$$

The above equations have trivial solutions at $\theta = 0$ and $\theta = \pi$. Stability conditions are now evaluated for each minimum independently.

Minimum A ($\theta = 0$):

$$\left.\begin{aligned}&\mu_0 M H t_1 + 2K_{\mathrm{ua}}\, t_1 > 0 \\ &(\mu_0 M H t_2 + K_{\mathrm{ud}}) + 2K_{\mathrm{ua2}}\, t_2 > 0\end{aligned}\right\} \mu_0 M H (t_1 + t_2) + K_{\mathrm{ud}} + 2K_{\mathrm{ua1}}\, t_1 + 2K_{\mathrm{ua2}}\, t_2 > 0 \tag{S6}$$

$$H > \frac{-K_{\mathrm{ud}} - 2K_{\mathrm{ua1}}\, t_1 - 2K_{\mathrm{ua2}}\, t_2}{\mu_0 M (t_1 + t_2)} \tag{S7}$$

Minimum B ($\theta = \pi$):

$$\left.\begin{aligned}&-\mu_0 M H t_1 + 2K_{\mathrm{ua}}\, t_1 > 0 \\ &-(\mu_0 M H t_2 + K_{\mathrm{ud}}) + 2K_{\mathrm{ua2}}\, t_2 > 0\end{aligned}\right\} -\mu_0 M H (t_1 + t_2) - K_{\mathrm{ud}} + 2K_{\mathrm{ua1}}\, t_1 + 2K_{\mathrm{ua2}}\, t_2 > 0 \tag{S8}$$

$$H < \frac{-K_{\mathrm{ud}} + 2K_{\mathrm{ua1}}\, t_1 + 2K_{\mathrm{ua2}}\, t_2}{\mu_0 M (t_1 + t_2)} \tag{S9}$$

Using the conventional definitions of the switching fields in EB systems $H_1 = H_{\mathrm{EB}} + H_{\mathrm{c}}$ and $H_2 = H_{\mathrm{EB}} - H_{\mathrm{c}}$, we can identify H_{EB} and H_{c} as:

$$H_{\mathrm{EB}} = \frac{-K_{\mathrm{ud}}}{\mu_0 M (t_1 + t_2)} \qquad H_{\mathrm{c}} = \frac{2K_{\mathrm{ua1}}\, t_1 + 2K_{\mathrm{ua2}}\, t_2}{\mu_0 M (t_1 + t_2)} \tag{S10}$$

We find that the EB field H_{EB} scales inversely with the sum of the thickness of both layers $t_1 + t_2$ in the limit of strongly coupled layers. The same EB field is also expected for a single EB layer with total thickness $t_1 + t_2$. The coercive field H_c is proportional to the thickness-weighted sum of the individual anisotropy constants for both layers. Assuming $K_{\mathrm{ua1}} = K_{\mathrm{ua2}} = K_{\mathrm{ua}}$ one also obtains the expected result for a single EB layer $H_{\mathrm{c}} = \frac{2K_{\mathrm{ua}}}{\mu_0 M}$, which is independent of the layer thickness.

For case (II) of fully decoupled layers we get:

$$\begin{aligned}\frac{\partial E_{\mathrm{tot}}}{\partial \theta_1} &= \mu_0 M H t_1 \sin(\theta_1) + 2K_{\mathrm{ua1}}\, t_1 \cos(\theta_1)\sin(\theta_1) = 0 \\ \frac{\partial E_{\mathrm{tot}}}{\partial \theta_2} &= (\mu_0 M H t_2 + K_{\mathrm{ud}}) \sin(\theta_2) + 2K_{\mathrm{ua2}}\, t_2 \cos(\theta_2)\sin(\theta_2) = 0\end{aligned} \tag{S11}$$

These equations can also be solved independently, with the solutions $\theta_1 = 0, \pi$ and $\theta_2 = 0, \pi$, respectively. The conditions for stable energy minima in this case read:

$$\begin{aligned}\frac{\partial^2 E_{\mathrm{tot}}}{\partial \theta_1^2} &= \mu_0 M H t_1 \cos(\theta_1) + 2K_{\mathrm{ua1}}\, t_1 \cos^2(\theta_1) - 2K_{\mathrm{ua1}}\, t_1 \sin^2(\theta_1) > 0 \\ \frac{\partial^2 E_{\mathrm{tot}}}{\partial \theta_2^2} &= (\mu_0 M H t_2 + K_{\mathrm{ud}}) \cos(\theta_2) + 2K_{\mathrm{ua2}}\, t_2 \cos^2(\theta_2) - 2K_{\mathrm{ua2}}\, t_2 \sin^2(\theta_2) > 0\end{aligned} \tag{S12}$$

Inserting the above solutions in the stability conditions for minima yields for θ_1:

$$\begin{aligned}&\theta_1 = 0 && \theta_1 = \pi \\ &\mu_0 M H t_1 + 2K_{\mathrm{ua}}\, t_1 > 0 && -\mu_0 M H t_1 + 2K_{\mathrm{ua}}\, t_1 > 0 \\ &H > -\frac{2K_{\mathrm{ua1}}}{\mu_0 M} && H < \frac{2K_{\mathrm{ua1}}}{\mu_0 M}\end{aligned} \tag{S13}$$

and for θ_2:

$$\begin{aligned}&\theta_2 = 0 && \theta_2 = \pi \\ &\mu_0 M H t_2 + K_{\mathrm{ud}} + 2K_{\mathrm{ua}}\, t_2 > 0 && -\mu_0 M H t_2 - K_{\mathrm{ud}} + 2K_{\mathrm{ua}}\, t_2 > 0 \\ &H > \frac{-K_{\mathrm{ud}} - 2K_{\mathrm{ua2}}\, t_2}{\mu_0 M t_2} && H < \frac{-K_{\mathrm{ud}} + 2K_{\mathrm{ua2}}\, t_2}{\mu_0 M t_2}\end{aligned} \tag{S14}$$

Using the definitions of swiching fields independently for both layers we obtain the result:

$$H_{\mathrm{EB1}} = H_{\mathrm{o}} = 0 \qquad H_{\mathrm{c1}} = \frac{2K_{\mathrm{ua1}}}{\mu_0 M} \tag{S15}$$

and

$$H_{\mathrm{EB2}} = -\frac{K_{\mathrm{ud}}}{\mu_0 M t_2} \qquad H_{\mathrm{c2}} = \frac{2K_{\mathrm{ua2}}}{\mu_0 M} \tag{S16}$$

For fully decoupled layers, only the hysteresis loop of the pinned layer has a non-zero EB shift, which is identical to the expected EB shift for a single EB layer with thickness t_2. Coercive fields in both pinned and top layers are independent of layer thickness and yield the expected results of the anisotropy fields for the respective layer. We compare our experimental results to the analytical predictions for both limiting case in Figure S3.

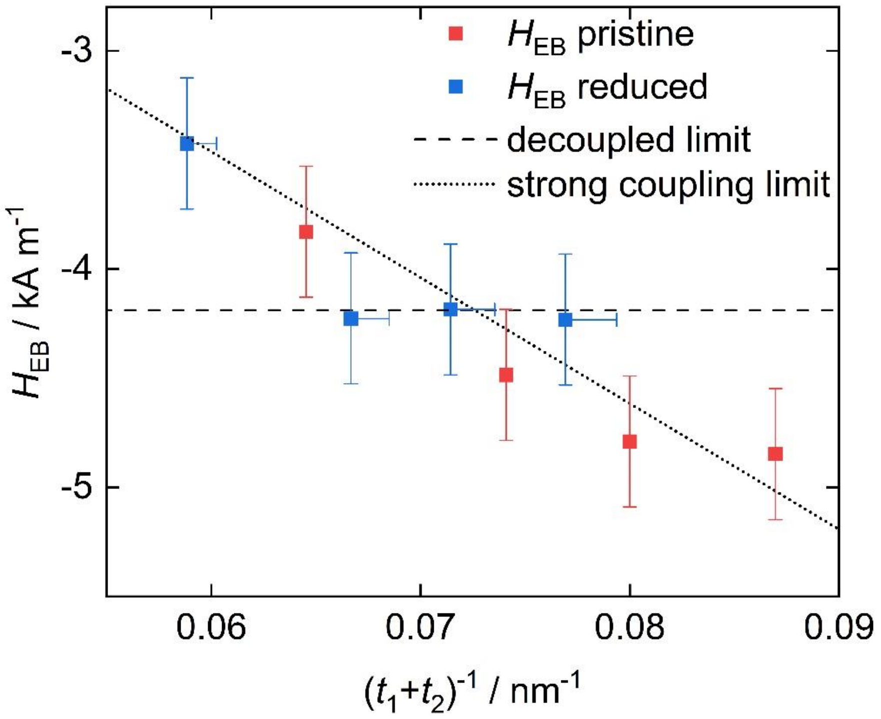

Figure S3. Comparison of experimental exchange bias fields H_{EB} with analytical expressions derived from the SW model in S3 for the limit of strong coupling (dotted line) and full decoupling (dashed line). All fields are plotted over inverse total thickness $(t_1+t_2)^{-1}$. The slope of the line for the strong coupling limit is given by $-K_{ud}/\mu_0 M_{Fe}$, where K_{ud} is chosen to be 1.24 $\cdot 10^{-4}$ J m^{-2} for the best fit with experimental data. The constant H_{EB} in the decoupled limit, is calculated from an (arbitrarily chosen) unidirectional anisotropy constant of K_{ud} = 0.90 $\cdot 10^{-4}$ J m^{-2}, the pinned Fe layer thickness t_2 = 10 nm, and the saturation magnetization of Fe. Note that exchange bias fields are plotted with a negative sign here to facilitate comparison with Reference 4.

If the interaction in our spin-valve heterostructure could be described either by the strong coupling or the fully decoupled limit, data for H_{EB} in both reduced and oxidized states should lie on a straight line in a plot over the total thickness. For the decoupled case a horizontal line (no thickness dependence) would be expected, while for the case of strong coupling a constant slope would be given by the expression for the EB field. As seen in Figure S3, H_{EB} as a function of thickness for our experimental curves roughly follows the prediction in the strong coupling limit. However, this simple model also predicts an unchanging coercivity H_c with top layer thickness, which we do not observe in our MI experiments. Instead, we use modeled hysteresis loops in the main text for a more accurate description of hysteresis loops and all corresponding characteristic fields.

S4. Atomic force microscopy images

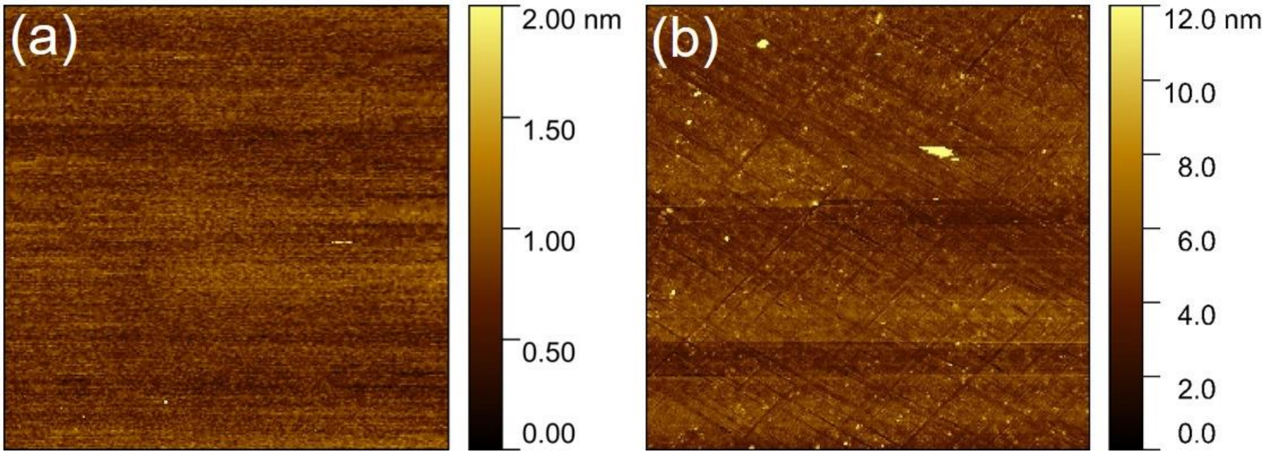

Figure S4. Atomic force microscopy (AFM) images (10 × 10 µm²) in tapping mode of a the IrMn/Fe/Au/Fe sample ($t_{top,nom}$ = 6 nm, t_{Au} = 5 nm) used for the switching experiment over 40 measurement steps in Figure 4 in the main text. In (a) an untreated part of the sample surface is shown, while in (b) an area that has been exposed to the electrochemical treatment is depicted. Note the different scalebars for height for both images. The images are recorded on a JPK Nanowizard II AFM system, processing uses the open-source software Gwyddion.[5] The RMS roughness over the full image increases from ~0.2 nm on the untreated area to ~1.5 nm after magneto-ionic treatment. Along selected single AFM line scans avoiding obvious surface defects, the RMS roughness in is ~0.12 nm the untreated state and ~0.5 nm after magneto-ionics, indicating the roughness increase is not due to a surface contamination, but due to a roughening of the top Fe layer.

S5. Coupling strength for Néel coupling as a function of top layer thickness

Offset fields of the top layer loop from hysteresis loops are commonly used to calculate coupling constants J using the equation

$$H_o = \frac{J}{\mu_0 M t_{top}}, \qquad \text{(S17)}$$

where M is the magnetization of the top layer, and t_{top} the top layer thickness. These coupling constants can then be compared to the expected coupling constants from structural parameters for the Néel coupling. However, this relation between offset field H_o and coupling constant J does not hold in the limit of strong coupling, when H_o can only be derived from J solving the SW model numerically. In our case, we can only compare the coupling constants used for modeling hysteresis loops in our SW model to the expected coupling constants for a Néel coupling from structural parameters.

The following expression has been used to calculate the coupling constant *J* for Néel coupling from structural parameters:[6]

$$J = \frac{\pi^2 h^2 \mu_0 M^2}{\sqrt{2}\lambda}\left[1 - exp\left(\frac{-2\pi\sqrt{2}t_{\mathrm{top}}}{\lambda}\right)\right] \times \left[1 - \exp\left(\frac{-2\pi\sqrt{2}t_{\mathrm{pinned}}}{\lambda}\right)\right]\exp\left(\frac{-2\pi\sqrt{2}t_{\mathrm{IL}}}{\lambda}\right), \quad \text{(S18)}$$

where *h* is the amplitude of the roughness oscillation, *λ* is the oscillation wavelength, t_{pinned} is the pinned layer thickness, and t_{IL} is the interlayer thickness. In our case we use $\sqrt{2}$ times the RMS roughness from AFM images as our value for *h*,[7] which is around 0.35 nm RMS on average for the sample series, and the grain size d from TEM images for *λ*, which was between 15 nm and 20 nm. In our coupled spin-valves t_{pinned} and t_{IL} are 10 nm and 4 nm, respectively. We plot *J* as a function of t_{top} for both grain sizes in Figure S5 and compare it to the *J* used for modeling our loops in Figure 3 in the main manuscript. Note that for a RKKY-type coupling a constant *J*, i.e. no pronounced thickness dependence as function of top layer thickness t_{top}, would be expected.[8]

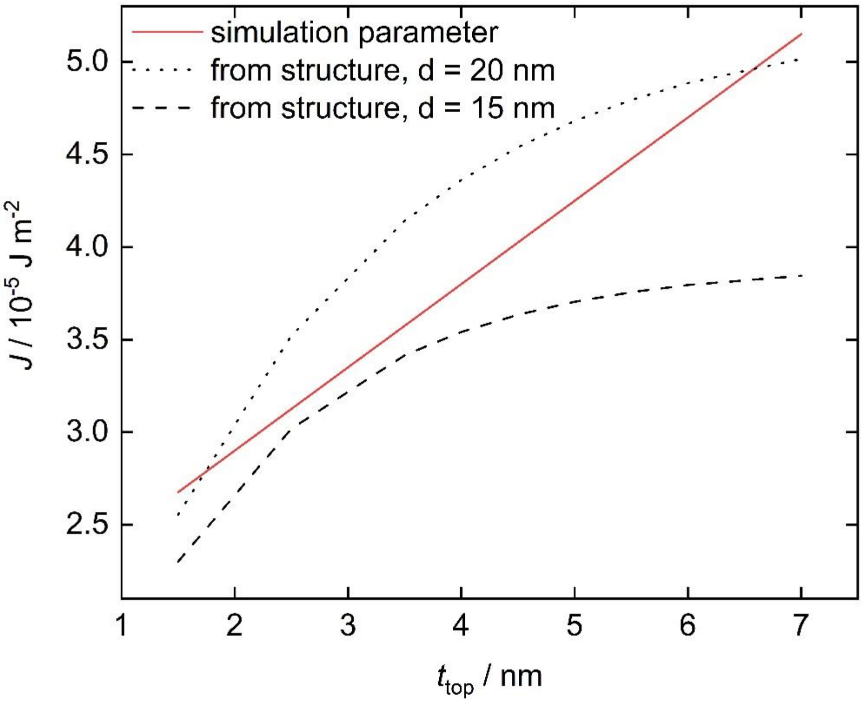

Figure S5. Coupling constant *J* estimated from the equation for Néel coupling compared with the coupling constants used to model hysteresis loops. Two different grain sizes d (15 and 20 nm) were used as λ in the equation for Néel coupling, which is the range of grain sizes from TEM images. While a linear increase in coupling constant was used for the modeling, the calculated coupling constants from structural parameters show a different increase, but are in the same order of magnitude required for a strong interlayer coupling.

S6. Biquadratic coupling energy

For biquadratic coupling the following expression has been proposed as a contribution to the total energy[9]:

$$E_{\mathrm{bq}} = J_2 \cos^2(\theta_1 - \theta_2) \quad , \tag{S19}$$

where J_2 is positive. For a parallel or antiparallel alignment of the pinned layer with the external field, which is the case for a sharp switching field of the pinned layer and a good approximation of our experimental hysteresis loops, we have $\theta_2 = 0$ or $\theta_2 = \pi$ for all fields H, which allows us to write the biquadratic coupling energy as:

$$E_{\mathrm{bq}} = J_2 \cos^2(\theta_1) \tag{S20}$$

Using elementary algebra, this expression can be transformed into:

$$E_{\mathrm{bq}} = -J_2 \cos^2(\theta_1 - \frac{\pi}{2}) + \mathrm{const.} \tag{S21}$$

This expression is mathematically equivalent to a term which we use for the anisotropy energy of the top layer:

$$E_{\mathrm{ua1}} = -K_{\mathrm{ua}} \cos^2(\theta_1 - \psi_1) \quad , \tag{S22}$$

where ψ_1 is the offset angle of the anisotropy axis from the unidirectional anisotropy (and field) direction. Without the explicit inclusion of an energy term for biquadratic coupling, in our model the biquadratic coupling energy can manifest itself as an extra contribution to the anisotropy energy. Specifically, large offset angles ($\psi_1 \rightarrow \frac{\pi}{2}$) could therefore be an indicator for a stronger biquadratic coupling in our samples.

S7. Modeling hysteresis loops from the SW model for $t_{top,nom}$ = 6 nm

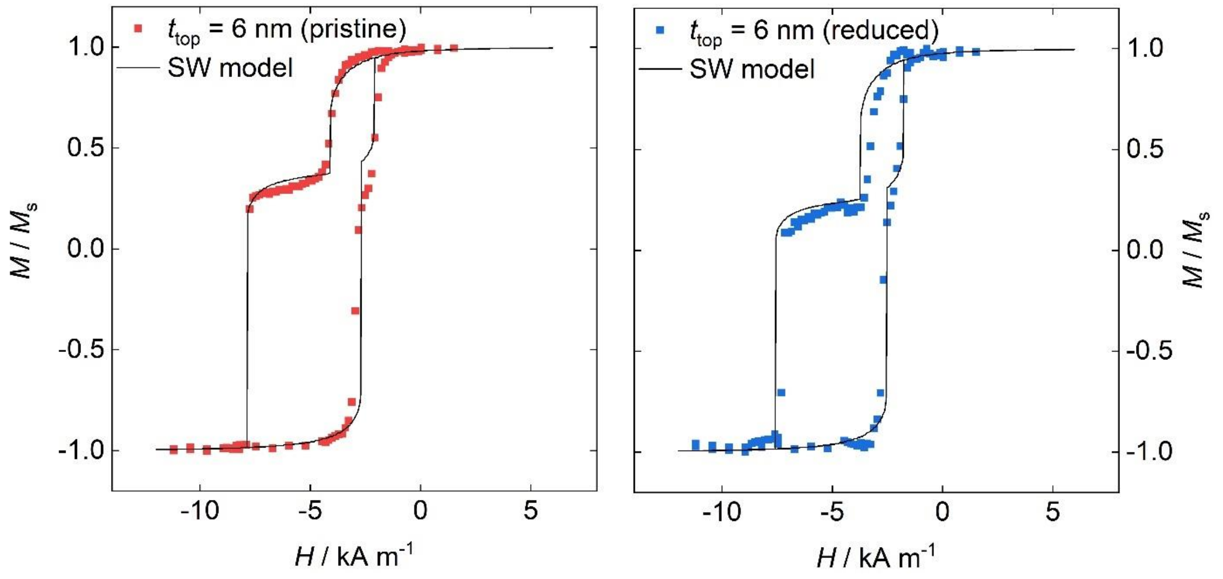

Figure S7. Comparison of experimental and modeled hysteresis loops in pristine (left) and reduced state (right). Experimental data points are drawn as red and blue squares.

For the modeling of hysteresis loops of the sample with a top layer thickness $t_{top,nom}$ = 6 nm, interlayer coupling strength and unidirectional (EB) anisotropy have been varied compared to model in the main text in Figure 3(b) to account for the stronger EB in this sample. In particular, we have used t_{top} = 4.5 nm, $K_{ud} = 1.4 \cdot 10^{-4}$ J m^{-2}, and $J = 3.2 \cdot 10^{-5}$ J m^{-2} for modeling of the pristine loops, while t_{top} = 6 nm, $K_{ud} = 1.4 \cdot 10^{-4}$ J m^{-2}, and $J = 3.8 \cdot 10^{-5}$ J m^{-2} have been used for the reduced state. The remaining parameters are identical compared to the model described in the main text, i.e. t_{pinned} = 10 nm, ψ_1 = 30°, ψ_2 = 15°, $K_{ua\ (pinned)}$ = 5.4 kJ m^{-3}, $K_{ua\ (top)}$ = 2.5 kJ m^{-3}, and $M = 1.71 \cdot 10^6$ A m^{-1}.

The exchange-bias field for the sample with $t_{top,nom}$ = 6 nm extracted from the hysteresis loops in Figure 1 is significantly larger compared to the sample with t_{top} = 5 nm (H_{EB} = 5.4 kA m^{-1} compared to H_{EB} = 4.5 kA m^{-1}), which we ascribe to a reduced thickness of the AFM layer as evidenced by XRR curves. The dependency of exchange-bias strength on the AFM thickness has been studied for AFM/FM bilayers.[10–12] It is known that for very thin AFM layers (< 10 nm) EB decreases with decreasing thickness, which does not reflect the change in H_{EB} for 6 nm sample compared to others in our case. However, the thickness of the AFM might also influence the EB field indirectly in polycrystalline layers, via a changing AFM grain size with layer thickness.

S8. Hysteresis loops parallel and perpendicular to the EB direction

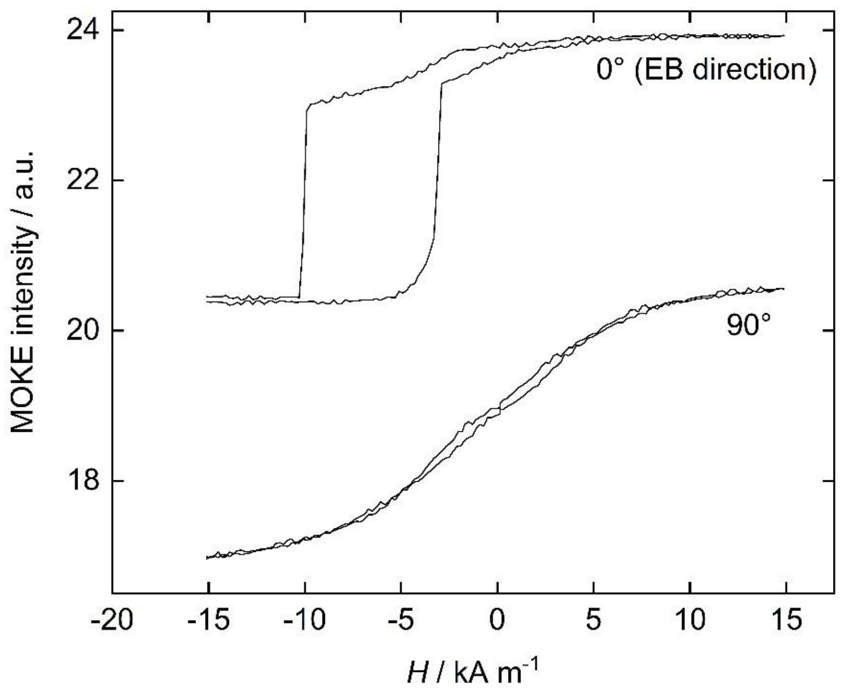

Figure S8. In-plane hysteresis loops of a Au(5 nm)/IrMn(30 nm)/Fe(10 nm)/Au(5 nm)/Fe(6 nm) heterostructure in the EB direction and perpendicular to the EB direction. A hard axis loop is observed at 90°, indicating that both Fe layers have an easy axis in or close to the EB direction.

S9. Hysteresis loops during stepwise reduction

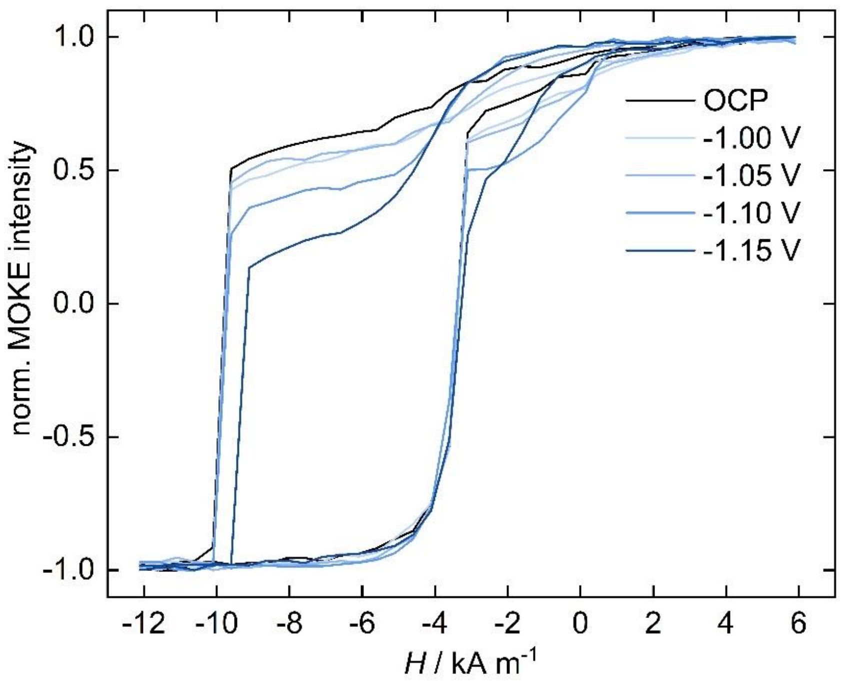

Figure S9. Hysteresis loops of a Au(5 nm)/IrMn(30 nm)/Fe(10 nm)/Au(5 nm)/Fe(6 nm) heterostructure in the EB direction as a function of applied potential. The potential is lowered in steps of 0.05 V starting at -1.00 V. A splitting into two separate loops is observed at the onset potential of E = -1.10 V.

S10. Hysteresis loops after potential switch-off under open circuit conditions

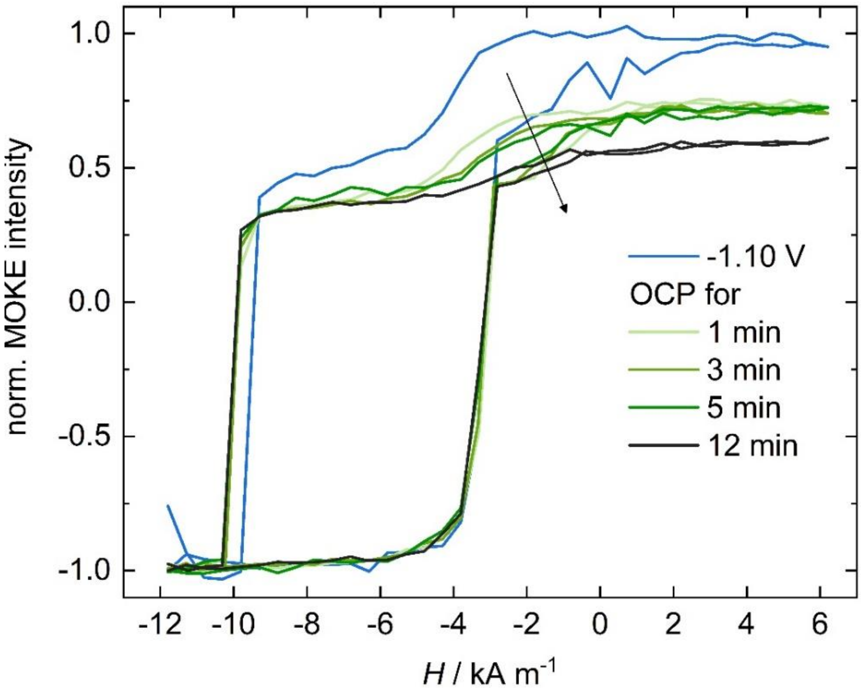

Figure S10. Hysteresis loops of a Au(5 nm)/IrMn(30 nm)/Fe(10 nm)/Au(5 nm)/Fe(6 nm) heterostructure in the EB direction after removal of a reduction potential over time. An initial reduction step at E = -1.10 V causes formation of a double-step loop, which gradually transforms into a single-step loop upon removal of the potential at the open circuit potential (OCP). This indicates that the reduced Fe layer in our heterostructures is spontaneously oxidized back to FeO_x in the electrolyte. The OCP was -0.07 V after 12 minutes. One should note that it takes longer to return to a full single-step loop under open circuit conditions, compared to the potentiostatic oxidation at E = -0.02 V shown in Figure 4, where a single-step loop is obtained in under a minute.

S11 and S12. Modeled hysteresis loops for t_{Au} = 5 nm, t_{top} = 6 nm

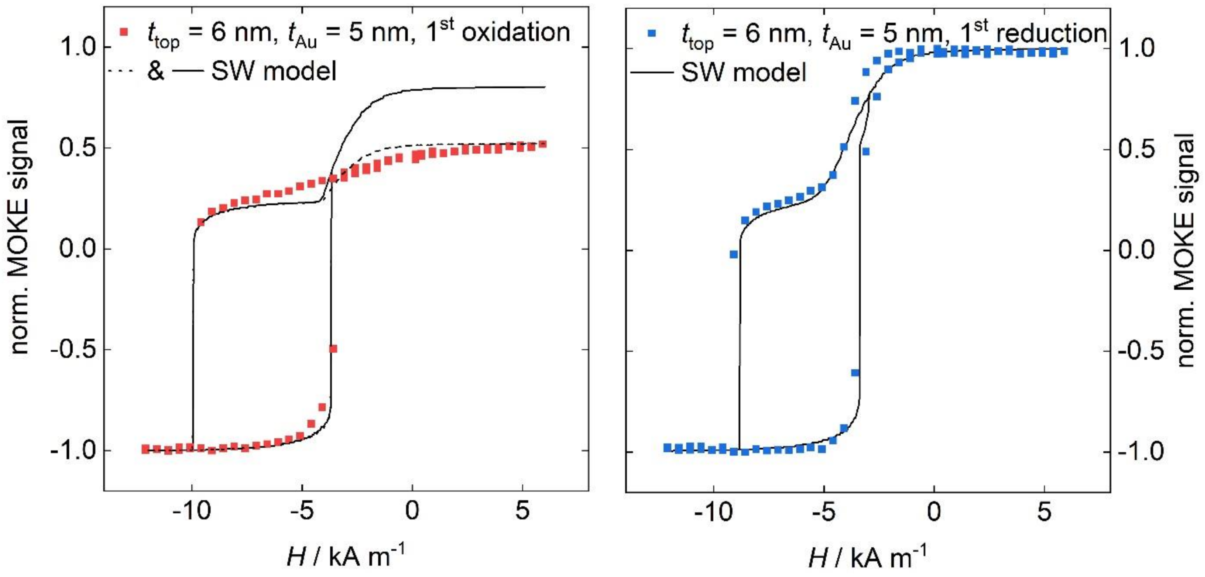

Figure S11. Comparison of experimental and modeled hysteresis loops for 1st oxidized (left) and 1st reduced state (right) for the spin-valve switching experiment on a Au(5 nm)/IrMn(30 nm)/Fe(10 nm)/Au(5 nm)/Fe(6 nm) heterostructure (Figure 4 in the main text). Experimental data points are drawn as red and blue squares. Full lines show hysteresis loops as obtained from the SW model, while the dashed line corresponds to a loop with a reduced top layer magnetization by a factor of 0.5. All loops are normalized using the saturation intensity in the reduced state.

The following parameters are used in the extended SW model for the loops spin-valve switching experiment of the Au(5 nm)/IrMn(30 nm)/Fe(10 nm)/Au(5 nm)/Fe(6 nm) heterostructure: t_{top} = 4.5 nm / 6 nm (oxidized / reduced state), J = 2.8·10^{-5} J m^{-2} / 4.6 ·10^{-5} J m^{-2} (oxidized / reduced state), K_{ud} = 1.7 ·10^{-4} J m^{-2}, t_{pinned} = 10 nm, ψ_1 = 60°, ψ_2 = 15°, $K_{ua\,(pinned)}$ = 6.2 kJ m^{-3}, $K_{ua\,(top)}$ = 1.1 kJ m^{-3}, and M = 1.71·10^6 A m^{-1}.

As discussed in S6, the larger angle of the uniaxial anisotropy axis for the top layer with the external field direction (ψ_1 = 60°) can be ascribed to a stronger contribution of biquadratic coupling. For the oxidized loop, the regular SW model is plotted as a full line, while a loop with a reduced top layer magnetization (by a factor of 0.5) is plotted as a dashed line. This could be the result of a rigid biquadratic coupling, which favors perpendicular alignment of magnetization in both layers. Such a coupling contribution could effectively turn the top layer into a hard axis configuration that prevents full saturation in measured field range. Preliminary tests for an extended SW model with an explicit form of the biquadratic energy included support this possibility (not shown). In experimental loops, however, a changing MOKE sensitivity (direction) would also

contribute to overall curve shape. Characteristic fields extracted from these modeled loops are shown as dashed lines in Figure 4 in the main text.

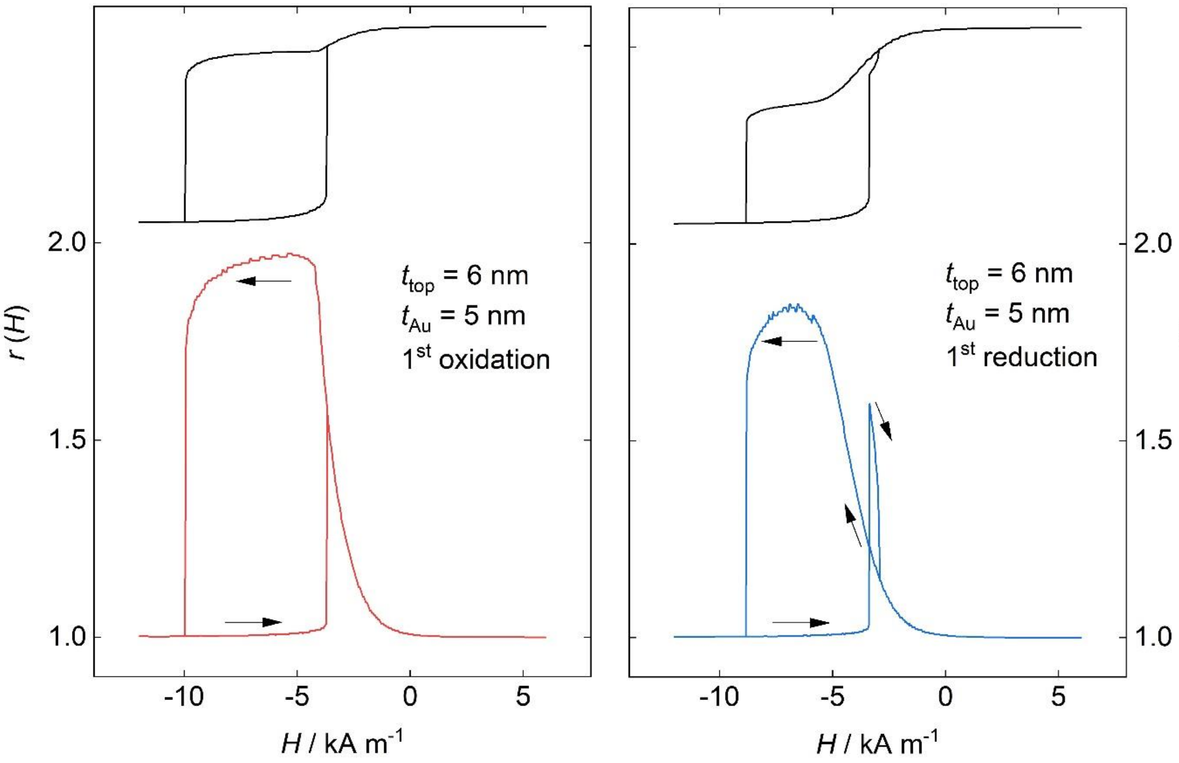

Figure S12. Calculated giant magnetoresistance (GMR) from the modeled hysteresis loops from Figure S11. Hysteresis loops are shown again on top of the GMR curves as a reference. Arrows indicate the GMR in the positive and negative field sweeps. The function *r* (*H*) is a dimensionless quantity, which corresponds to the normalized magnetoresistance for a parallel (*r* (*H*) = 1) and an antiparallel (*r* (*H*) = 2) layer alignment. Magnetoresistance can be calculated from the magnetization angles θ_1 and θ_2 in the SW model as:[13] $r(H) = 1 + \frac{1}{2}\{1 - \cos[\theta_1(H) - \theta_2(H)]\}$. The sharp spike in *r* (*H*) on the right indicates a sensitive GMR in a narrow field range for the modeled loop after reduction, which is absent in the modeled loop for the oxidized state.

S13. Modeling coupled spin valve hysteresis loops with antiferromagnetic coupling

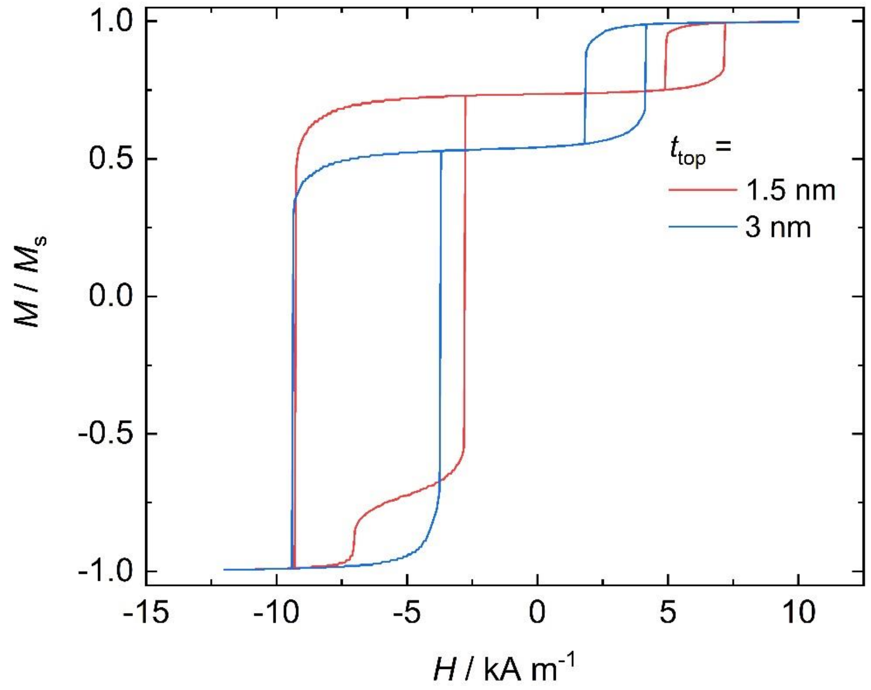

Figure S13. Modeled hysteresis loops for a Au/IrMn/Fe/Au/Fe heterostructure with two different top layer thicknesses t_{top} assuming a hypothetical antiferromagnetic interlayer coupling through the Au layer. Although such an antiferromagnetic coupling has not been found in our experiments, this demonstrates that a modification of top layer thickness, e.g. by using our magneto-ionic mechanism of FeO_x reduction to Fe, could also lead to a pronounced variation of exchange bias in antiferromagnetically coupled spin-valve heterostructures. We used the same model parameters for the calculation of hysteresis as a function of top layer thickness as in the main text, with the only difference that a fixed negative J was used for an antiferromagnetic coupling. The full parameter set was: t_{top} = 1.5 nm / 3 nm (oxidized / reduced state), $J = -2.0\cdot10^{-5}$ J m^{-2}, $K_{ud} = 1.2 \cdot10^{-4}$ J m^{-2}, t_{pinned} = 10 nm, ψ_1 = 30°, ψ_2 = 15°, $K_{ua\ (pinned)}$ = 5.4 kJ m^{-3}, $K_{ua\ (top)}$ = 2.5 kJ m^{-3}, and $M = 1.71\cdot10^6$ A m^{-1}.

References

1. Glavic, A. & Björck, M. *GenX 3* : the latest generation of an established tool. *J Appl Crystallogr* **55**, 1063–1071 (2022).

2. Zehner, J. *et al.* Robust Magneto-Ionic Effect in Fe/FeO $_x$ Thin Films in Electrolytes With Different Cations. *IEEE Trans. Magn.* **58**, 1–8 (2022).

3. Duschek, K., Uhlemann, M., Schlörb, H., Nielsch, K. & Leistner, K. Electrochemical and in situ magnetic study of iron/iron oxide films oxidized and reduced in KOH solution for magneto-ionic switching. *Electrochem. Commun.* **72**, 153–156 (2016).

4. Zehner, J. *et al.* Nonvolatile Electric Control of Exchange Bias by a Redox Transformation of the Ferromagnetic Layer. *Adv. Electron. Mater.* **5**, 1900296 (2019).

5. Nečas, D. & Klapetek, P. Gwyddion: an open-source software for SPM data analysis. *Open Physics* **10**, 181–188 (2012).

6. Kools, J. C. S., Kula, W., Mauri, D. & Lin, T. Effect of finite magnetic film thickness on Néel coupling in spin valves. *Journal of Applied Physics* **85**, 4466–4468 (1999).

7. Parks, D. C., Chen, P. J., Egelhoff, W. F. & Gomez, R. D. Interfacial roughness effects on interlayer coupling in spin valves grown on different seed layers. *Journal of Applied Physics* **87**, 3023–3026 (2000).

8. Opitz, J., Zahn, P., Binder, J. & Mertig, I. *Ab initio*calculation of the interlayer exchange coupling in Fe/Au multilayers: The role of impurities at the interface. *Phys. Rev. B* **63**, (2001).

9. Slonczewski, J. C. Origin of biquadratic exchange in magnetic multilayers (invited). *Journal of Applied Physics* **73**, 5957–5962 (1993).

10. Merkel, M., Reginka, M., Huhnstock, R. & Ehresmann, A. Polycrystalline exchange-biased bilayers: Magnetically effective versus structural antiferromagnetic grain volume distribution. *Phys. Rev. B* **106**, 014403 (2022).

11. Ali, M. *et al.* Antiferromagnetic layer thickness dependence of the IrMn/Co exchange-bias system. *Phys. Rev. B* **68**, 214420 (2003).

12. Hu, J., Jin, G. & Ma, Y. Thickness dependence of exchange bias and coercivity in a ferromagnetic layer coupled with an antiferromagnetic layer. *Journal of Applied Physics* **94**, 2529–2533 (2003).

13. Rijks, Th. G. S. M., Coehoorn, R., Daemen, J. T. F. & De Jonge, W. J. M. Interplay between exchange biasing and interlayer exchange coupling in Ni80Fe20/Cu/Ni80Fe20/Fe50Mn50 layered systems. *Journal of Applied Physics* **76**, 1092–1099 (1994).
